# Fixed or Random Effects: Analysis of Clustered Data

**DOI:** 10.1002/sim.70687

**Published:** 2026-08-06

**Authors:** Kevin He, Xiangeng Fang, Yubo Shao, Nicholas Hartman, John D. Kalbfleisch

**Affiliations:** ^1^ Department of Biostatistics University of Michigan Ann Arbor Michigan USA; ^2^ Kidney Epidemiology and Cost Center University of Michigan Ann Arbor Michigan USA

**Keywords:** between and within models, biased estimation, provider profiling, simulation

## Abstract

In analyzing clustered data, random effects (RE) and fixed effects (FE) models are two primary approaches. The RE model assumes that the cluster‐specific effects are random and uncorrelated with individual characteristics. If this assumption is violated, the RE approach can lead to biased estimates of the coefficients of individual covariates and cluster effects. In contrast, the FE approach, which models cluster effects as fixed effects, yields unbiased estimates even if the cluster effects are correlated with individual covariates. Numerous studies have compared the FE and RE approaches. However, the practical importance of the biases observed in RE‐based methods remains unclear. Some studies have demonstrated significant biases in RE models when the assumption of no correlation is violated, whereas others have found these biases to be negligible. These differences may be due to variations in study design, with some evaluations focusing on settings that may not reflect realistic data scenarios. In this paper, we compare FE and RE models through theoretical evaluation, simulation studies, and real‐data applications. Specifically, we derive an approximate formula to quantify the asymptotic bias in the RE model and verify its accuracy through simulations. These results help to explain the different conclusions reached in the existing literature. Our goal is not to propose a new estimator or a fundamentally new inferential framework, but to provide an analytical clarification and practical quantification of the bias incurred by the standard RE estimator under a specific, interpretable dependence model, and to clarify how this bias relates to fixed‐effects and correlated random‐effects/between‐within specifications.

## 1 Introduction

In biomedical studies and social science research, clustered or grouped data are commonly observed due to the hierarchical nature of study units, such as students within schools [1, 2, 3, 4], patients within healthcare providers [5, 6, 7, 8, 9, 10], or employees within companies [11]. When analyzing clustered data, there are two primary methods to model cluster effects: random effects (RE) and fixed effects (FE).

With an RE approach, the cluster effects are modeled as independent and identically distributed random variables, often assuming a normal distribution. This method typically relies on the empirical Bayes method to determine the “posterior” distribution of the random effect for each cluster. The effect of each cluster is then estimated using the posterior mean. The RE analysis yields “shrinkage” estimates, in which the estimated cluster effects are shrunk towards the overall average effect. Note that an important assumption for the RE analysis (called the “RE assumption”) is that the cluster effects are independent of individual covariates. This assumption is rarely satisfied, but is often ignored in applications. As a result, the confounding between the effects of the cluster and the individual covariates can lead to substantially biased estimates of their coefficients and can alter the estimates of the cluster effects [9, 12].

In an FE approach, the cluster effects are considered as fixed effects. FE‐based estimates of the regression effects and the cluster effects are unbiased even if the cluster effects are correlated with individual covariates. In this way, the FE analysis is more robust than the RE analysis [9, 12]. On the other hand, if the cluster effects are independent of the covariates, the RE analysis and the corresponding shrinkage estimates reduce the marginal mean squared error (MSE) of the estimates for the coefficients and cluster effects. This result has motivated many authors to use RE methods in cluster data analysis [5, 6, 8, 13]. Although the RE approach achieves an overall reduction in MSE, this is at the cost of significant increases in conditional MSE to estimate cluster effects that are extreme. These extreme effects are particularly important in certain applications, such as healthcare provider profiling, which involves evaluating the performance of healthcare providers by identifying outliers or significant deviations from expected standards. This fact has led to compelling arguments that, at least in these areas, FE methods are preferable [9, 10].

Based on the existing literature, the degree to which the FE‐ and RE‐based approaches differ in estimating covariate effects is less clear. Hausman [14] observed notable differences in covariate estimates when applying the FE and RE models to the Michigan Income Dynamics study, which used data from 629 high school graduates [14]. Kalbfleisch and Wolfe [9] empirically showed that the RE approach can lead to substantially biased estimators when the effects of the cluster and the individual characteristics are correlated [9]. On the other hand, Clark and Linzer [15] conducted a series of simulations and found that the magnitude of the bias associated with the RE approach was typically small and generally not of practical importance [15]. This paper has been widely cited and used to justify RE analyses without regard for the validity of the RE assumption. Given these divergent conclusions, there is still no consistent guidance for researchers in choosing between FE or RE approaches.

To fill this gap, we compare the FE and RE models through both theoretical evaluation and comprehensive numerical experiments. Specifically, we derive an approximate formula to quantify bias in the RE model, which uncovers the differences between the approaches of Kalbfleisch and Wolfe [9] and Clark and Linzer [15]. The accuracy of this theoretical formula is validated through simulations, which also illustrate the magnitude of the bias in various scenarios. We do not claim to introduce a fundamentally new modeling or inferential framework. Rather, our contribution is to provide a focused analytical clarification and practical quantification of the bias of the standard RE estimator under a specific, interpretable dependence model, and to show when this bias becomes practically consequential under realistic study designs. Although previous work, such as Neuhaus and Kalbfleisch [16] and Shen et al. [17], has explored the asymptotic behavior of RE estimators when covariate effects can be decomposed into between‐ and within‐cluster components, our framework makes explicit how the standard RE bias depends on cluster size, signal‐to‐noise ratio, and the decomposition of covariate variation into within‐ and between‐cluster components. The framework includes the study designs considered in Kalbfleisch and Wolfe [9] and Clark and Linzer [15] as special cases, and therefore clarifies the practical implications of violations of the RE assumption.

A substantial literature has developed methods that relax the standard RE assumption by decomposing covariates into between‐ and within‐cluster components. The correlated random effects (CRE), between‐within (BW), and related conditional‐likelihood approaches include cluster‐level covariate means and within‐cluster deviations as separate predictors, allowing the within‐cluster coefficient to be estimated consistently when the relevant contextual means are correctly specified Mundlak [18], Neuhaus and Kalbfleisch [16], Neuhaus and McCulloch [19], Shen et al. [17]. These methods are central to the analysis of clustered data with endogenous cluster effects and are used in our applications below. The focus of the present paper is complementary: we study the magnitude and practical consequences of the bias incurred by the standard RE estimator when this decomposition is not used, or when the relevant between‐cluster structure is only partially specified.

## 2 Review of Cluster Effect Estimators

Let $Y_{ij}$ represent a continuous outcome for subject j in cluster i, j=1,…,n, i=1,…,m, where n is the sample size for each cluster, and m is the number of clusters. Note that, to simplify the notation, we assume that the sample sizes across clusters are the same. Consider a linear regression model,

(1) $$Y_{ij}=\mu +\alpha_{i}+{\text{X}}_{ij}^{⊤}\beta +\epsilon_{ij},$$

where μ is the population mean, $\alpha_{i}$ is the cluster effect, $\epsilon_{ij}$ is the independently and identically distributed random noise with mean 0 and variance $\sigma_{\epsilon }^{2}$, ${\text{X}}_{ij}$ is a p‐dimensional vector of random variables of individual characteristics, and the regression coefficients, β, measure the within‐cluster relationship between the covariates and the response.

### 2.1 The Fixed Effects Estimator

In the FE approach, μ and $\alpha_{i}$ are treated as fixed parameters. Without additional constraints, μ and $\alpha_{i}$ are not separately identifiable. This can be addressed by introducing a constraint, for example, $\sum_{i=1}^{m}\alpha_{i}=0$. Alternatively, we can define $\zeta_{i}=\mu +\alpha_{i}$ for i=1,…,m, and then estimate the joint effects $\zeta_{i}$. The corresponding FE‐based ordinary least squares (OLS) objective function is given by

(2) $$S(\vartheta )=\overset{m}{\underset{i=1}{\sum }}\overset{n}{\underset{j=1}{\sum }}{\left(Y_{ij}-\zeta_{i}-{\text{X}}_{ij}^{⊤}\beta \right)}^{2},$$

where $\vartheta ={\left(\zeta^{⊤},\beta^{⊤}\right)}^{⊤}$ and $\zeta ={\left(\zeta_{1},\ldots ,\zeta_{m}\right)}^{⊤}$. A common approach in the literature is to treat ζ as nuisance parameters and eliminate them using techniques such as profile likelihood or conditional likelihood. Specifically, for given β, the estimate of $\zeta_{i}$ is ${\hat{\zeta }}_{i}={\bar{Y}}_{i}-{\bar{\text{X}}}_{i}^{⊤}\beta$, where ${\bar{Y}}_{i}=\sum_{j=1}^{n}Y_{ij}/n$ and ${\bar{\text{X}}}_{i}=\sum_{j=1}^{n}{\text{X}}_{ij}/n$. Setting $\zeta_{i}={\hat{\zeta }}_{i}$, i=1,…,m, in (2), the resulting objective function for β, denoted as S(β), is given by

$$S(\beta )=\overset{m}{\underset{i=1}{\sum }}\overset{n}{\underset{j=1}{\sum }}{\left((Y_{ij}-{\bar{Y}}_{i})-{\left({\text{X}}_{ij}-{\bar{\text{X}}}_{i}\right)}^{⊤}\beta \right)}^{2}.$$

It follows that

(3) $$\begin{array}{ll} {\hat{\beta }}_{\text{FE}} & ={\left(\overset{m}{\underset{i=1}{\sum }}\overset{n}{\underset{j=1}{\sum }}({\text{X}}_{ij}-{\bar{\text{X}}}_{i}){\left({\text{X}}_{ij}-{\bar{\text{X}}}_{i}\right)}^{⊤}\right)}^{-1}\\ & \;\times \left(\overset{m}{\underset{i=1}{\sum }}\overset{n}{\underset{j=1}{\sum }}({\text{X}}_{ij}-{\bar{\text{X}}}_{i})(Y_{ij}-{\bar{Y}}_{i})\right). \end{array}$$

Note that ${\hat{\beta }}_{\text{FE}}$ is the best linear unbiased estimator (BLUE) of the true parameter β, given the properties of $\epsilon_{ij}$ in (1) and the assumption that μ and $\alpha_{i}$ are fixed unknown parameters. Furthermore, ${\hat{\beta }}_{\text{FE}}$ is also consistent when n or m goes to infinity. Finally, the FE‐based estimator for $\zeta_{i}$ is

(4) $${\hat{\zeta }}_{\text{FE},i}={\bar{Y}}_{i}-{\bar{\text{X}}}_{i}^{⊤}{\hat{\beta }}_{\text{FE}},\;i=1,\ldots ,m.$$

### 2.2 The Random Effects Estimator

In the linear model (1), RE procedures treat $\alpha_{i}$'s as independent random variables. The classical approach assumes that, given $X={\left(X_{1}^{⊤},\ldots ,X_{m}^{⊤}\right)}^{⊤}$ with ${\text{X}}_{i}={\left[{\text{X}}_{i1},\ldots ,{\text{X}}_{in}\right]}^{⊤}$, the random effects are independent and identically distributed with mean 0 and variance $\sigma_{\alpha }^{2}$, typically with $\alpha_{i}|X\overset{i.i.d}{∼}𝒩(0,\sigma_{\alpha }^{2})$. This model is often written without noting the important RE assumption that the $X_{ij}$'s and $\alpha_{i}$ are independent.

Under the RE assumption, the FE‐based estimator in (3) is still unbiased and consistent when n or m goes to infinity. However, this FE estimator is not the BLUE. In accordance with the Gauss‐Markov theorem, assuming $\sigma_{\alpha }^{2}$ and $\sigma_{\epsilon }^{2}$ are known, the BLUE under the RE assumption is the RE estimator. Following Scott and Holt [20], the RE estimator for β can be represented as

(5) $$\begin{array}{ll} {\hat{\beta }}_{\text{RE}} & ={\hat{\beta }}_{W}+\lambda (\eta )({\hat{\beta }}_{B}-{\hat{\beta }}_{W}), \end{array}$$

where ${\hat{\beta }}_{B}$ is the between‐cluster estimator given by

$${\hat{\beta }}_{B}={\left(\overset{m}{\underset{i=1}{\sum }}({\bar{\text{X}}}_{i}-\bar{\text{X}}){\left({\bar{\text{X}}}_{i}-\bar{\text{X}}\right)}^{⊤}\right)}^{-1}\left(\overset{m}{\underset{i=1}{\sum }}({\bar{\text{X}}}_{i}-\bar{\text{X}}){\left({\bar{Y}}_{i}-\bar{Y}\right)}^{⊤}\right),$$

with $\bar{X}=\sum_{i=1}^{m}\sum_{j=1}^{n}X_{ij}/(mn)$ being the sample mean of the predictors, and $\bar{Y}=\sum_{i=1}^{m}\sum_{j=1}^{n}Y_{ij}/(mn)$ being the sample mean of the outcomes, ${\hat{\beta }}_{W}$ is the within‐cluster estimator, which is equivalent to the unbiased FE estimate in (3), η is the intra‐cluster correlation coefficient of $Y_{ij}$

(6) $$\eta =\frac{\sigma_{\alpha }^{2}}{\sigma_{\alpha }^{2}+\sigma_{\epsilon }^{2}},$$

and

(7) $$\lambda (\eta )=\frac{(1-\eta )\hat{\tau }}{\left(1-\eta )+n\eta (1-\hat{\tau }\right)}$$

with

$$\hat{\tau }={\left(\overset{m}{\underset{i=1}{\sum }}\overset{n}{\underset{j=1}{\sum }}({\text{X}}_{ij}-\bar{\text{X}}){\left({\text{X}}_{ij}-\bar{\text{X}}\right)}^{⊤}\right)}^{-1}\left(\overset{m}{\underset{i=1}{\sum }}n({\bar{\text{X}}}_{i}-\bar{\text{X}}){\left({\bar{\text{X}}}_{i}-\bar{\text{X}}\right)}^{⊤}\right).$$

Thus, ${\hat{\beta }}_{\text{RE}}$ is a weighted average of the between‐cluster and the within‐cluster estimators. The population intercept μ can then be estimated by

$${\hat{\mu }}_{\text{RE}}=\bar{Y}-{\bar{\text{X}}}^{⊤}{\hat{\beta }}_{\text{RE}}.$$

Finally, an empirical Bayes approach [21] gives the Best Linear Unbiased Predictor (BLUP) of the realized values of the random effect $\alpha_{i}$,

$${\hat{\alpha }}_{i}=\frac{\sigma_{\alpha }^{2}}{\sigma_{\alpha }^{2}+\sigma_{\epsilon }^{2}/n}\left({\bar{Y}}_{i}-{\hat{\mu }}_{\text{RE}}-{\bar{\text{X}}}_{i}^{⊤}{\hat{\beta }}_{\text{RE}}\right).$$

Remark 1 When $\alpha_{i}$ is independent of ${\text{X}}_{i}$ (the RE assumption is satisfied), the RE estimator (5) is unbiased [22] and consistent when n or m goes to infinity. However, when $\alpha_{i}$ is correlated with ${\text{X}}_{i}$ as in many clustered data applications [9], the RE estimator of β can be biased. More details are provided in Section 3.

Remark 2 In practice, $\sigma_{\alpha }^{2}$ and $\sigma_{\epsilon }^{2}$ are usually unknown. Alternatively, the unknown covariance parameters can be replaced by their consistent estimators, such as the maximum likelihood estimator (MLE) or the Restricted Maximum Likelihood estimator (REML). Further methodological details can be found in Demidenko [23]. For large samples, the differences between these methods become negligible.

## 3 A Framework to Assess Bias in the RE Approach

To assess the bias of the RE approach when $\alpha_{i}$ and $X_{ij}$ are correlated, we first consider the case with p=1 and an underlying model specified as follows: The variable $\gamma_{i}$ (the underlying within‐cluster mean of $X_{ij}$) is correlated with $\alpha_{i}$, and they are independent and identically distributed bivariate normal variables,

(8) $$\left(\begin{array}{l} \alpha_{i}\\ \gamma_{i} \end{array}\right)\overset{i.i.d}{∼}𝒩\left(\begin{array}{l} \left(\begin{array}{l} 0\\ 0 \end{array}\right),\left(\begin{array}{ll} \sigma_{\alpha }^{2} & \rho \sigma_{\alpha }\sigma_{\gamma }\\ \rho \sigma_{\alpha }\sigma_{\gamma } & \sigma_{\gamma }^{2} \end{array}\right) \end{array}\right),$$

and

(9) $$X_{ij}|\gamma_{i},\alpha_{i}\overset{i.i.d}{∼}𝒩(\gamma_{i},\sigma_{X}^{2}).$$

In this, ρ is the correlation between $\alpha_{i}$ and $\gamma_{i}$, and $\sigma_{X}^{2}$ is the conditional variance of $X_{ij}$ given $\gamma_{i}$ and $\alpha_{i}$. In what follows, we refer to this underlying model for (1) specified by (8) and (9) as TDM, meaning “The Dependence Model.”

Proposition 1 summarizes the magnitude of the asymptotic bias for the RE estimate of β when TDM is the true model and η is known. The proof is provided in the Supporting Information.

Proposition 1 *Consider the simple linear regression model* (*1*) *and assume that the data actually arise from the underlying model, TDM. Then, for any given* η, *the asymptotic bias in the RE estimator of* β *is*

(10) $$\begin{array}{ll} \underset{m\to \infty }{\lim}({\hat{\beta }}_{\text{RE}}-\beta ) & =\frac{(1-\eta )\rho \sigma_{\alpha }\sigma_{\gamma }}{(1-\eta )(\sigma_{\gamma }^{2}+\sigma_{X}^{2})+(n-1)\eta \sigma_{X}^{2}}\\ & =\frac{\rho \sigma_{\alpha }\sigma_{\gamma }}{\sigma_{X}^{2}}{\left\{1+\sigma_{\gamma }^{2}/\sigma_{X}^{2}+(n-1)\sigma_{\alpha }^{2}/\sigma_{\epsilon }^{2}\right\}}^{-1}. \end{array}$$

The bias expression in (10) is closely related to the probability‐limit calculation for the RE estimator in Neuhaus and McCulloch [19, eq (11)], and we thank the reviewer for pointing out this connection. In their less general marginal linear mixed model, the RE bias term can be written as

$$\begin{array}{l} \frac{\sigma_{xb}}{\sigma_{x}^{2}}\frac{\sigma_{\epsilon }^{2}}{\sigma_{\epsilon }^{2}+(n-1)\sigma_{b}^{2}}, \end{array}$$

where $\sigma_{xb}$ is the covariance between the covariate and the random intercept, and $\sigma_{x}^{2}$ is the marginal variance of the covariate. Under the present TDM, $\mathrm{Cov}(X_{ij},\alpha_{i})=\rho \sigma_{\alpha }\sigma_{\gamma }$ and $\mathrm{Var}(X_{ij})=\sigma_{\gamma }^{2}+\sigma_{X}^{2}$, but the explicit decomposition $X_{ij}=\gamma_{i}+\omega_{ij}$, with $\omega_{ij}$ independently and identically following a distribution $𝒩(0,\sigma_{X}^{2})$, induces within‐cluster correlation in the covariate and yields the denominator in (10), namely

$$\begin{array}{l} \sigma_{\epsilon }^{2}(\sigma_{\gamma }^{2}+\sigma_{X}^{2})+(n-1)\sigma_{\alpha }^{2}\sigma_{X}^{2}, \end{array}$$

after multiplying the numerator and denominator by $\sigma_{\epsilon }^{2}\sigma_{X}^{2}$. Thus, (10) should be viewed as a TDM‐specific refinement of the same general RE bias phenomenon, with the advantage that it separates the roles of within‐cluster covariate variation and between‐cluster covariate heterogeneity.

Remark 3 The magnitude of the asymptotic bias increases linearly with ρ. If the ratio $\sigma_{\alpha }\sigma_{\gamma }/\sigma_{X}^{2}$ is fixed, the bias decreases as $\sigma_{\alpha }^{2}/\sigma_{\epsilon }^{2}$, the signal‐to‐noise ratio (i.e., the ratio of the between‐cluster variation to the within‐cluster variation) for $Y_{ij}$, increases. Finally, the bias decreases as n increases and approaches 0 as n→∞. Notably, the bias does not depend on β.

Corollary 1 *The bias and MSE of* ${\hat{\beta }}_{\text{RE}}$ *do not depend on the true value of* β.

Remark 4 Proposition 1 examines asymptotic biases of the RE estimator when the intra‐cluster correlation, η, is known. However, in practice, η is typically unknown and must be estimated. As highlighted by Shen et al. [17], the estimated η is biased and exhibits complex behavior when the RE assumption is violated. In Section 4.3, we demonstrate the impact of estimating η compared to using a predetermined value.

In practice, the pooled OLS estimator of β is also sometimes used, and is generated from the following linear model obtained by setting $\alpha_{i}=0$ for all i in (1),

$$Y_{ij}=\mu +{\text{X}}_{ij}\beta +\epsilon_{ij}.$$

The corresponding pooled OLS estimator is given by

(11) $$\begin{array}{ll} {\hat{\beta }}_{\text{pooled}}= & {\left(\overset{m}{\underset{i=1}{\sum }}{\left({\text{X}}_{i}-\bar{\text{X}}\right)}^{⊤}({\text{X}}_{i}-\bar{\text{X}})\right)}^{-1}\left(\overset{m}{\underset{i=1}{\sum }}{\left({\text{X}}_{i}-\bar{\text{X}}\right)}^{⊤}(Y_{i}-\bar{Y})\right). \end{array}$$

Corollary 2 below shows that, like the RE estimator, the pooled OLS estimator can lead to biased estimation of β when $\alpha_{i}$ and ${\text{X}}_{i}$ are correlated.

Corollary 2 *Under the TDM model, the asymptotic bias* (m→∞) *of the pooled OLS estimator of* β *is*

$$\begin{array}{l} \frac{\rho \sigma_{\gamma }\sigma_{\alpha }}{\sigma_{\gamma }^{2}+\sigma_{X}^{2}}. \end{array}$$

Remark 5 When $(n-1)\sigma_{\alpha }^{2}/\sigma_{\epsilon }^{2}$ is small, the bias formula for the RE model approximates that for the pooled model. Note that the asymptotic bias of the pooled estimator does not depend on n.

Corollary 3 extends Proposition 1 to a multivariate scenario.

Corollary 3 *Consider the linear regression model*,

(12) $$Y_{ij}=\mu +\alpha_{i}+X_{ij}\beta +Z_{ij}^{⊤}\theta +\epsilon_{ij},$$

*where, as before*, $X_{ij}$ *is a single normal random variable that is correlated with* $\alpha_{i}$ (*i.e., endogenous variable*), *and* $Z_{ij}$ *is a vector of normally distributed covariates uncorrelated with* $\alpha_{i}$ (*i.e., exogenous variables*) *but potentially correlated with* $X_{ij}$ *. Again, we assume that* η *is known*.

*a*. *If* $Z_{ij}$ *is independent of* $X_{ij}$ *and* $\alpha_{i}$, *the RE estimate of* θ *is unbiased and the asymptotic bias in the estimate of* β *is given by* (*10*).
*b*. *If* $Z_{ij}$ *is correlated with* $X_{ij}$, *the RE estimate of* β *has an asymptotic bias given by* (*10*) *but with* $\sigma_{X}^{2}$ *replaced with* $\sigma_{X}^{*2}=\mathrm{Var}(X_{ij}|Z_{ij})$.

In addition to providing these new insights, (10) may serve as a practical tool for medical statisticians to guide their analytical choices with clustered data. For example, in applications with many clusters and/or covariates where the FE or CRE approaches are infeasible or less convenient to implement, investigators may plug in realistic values for the parameters involved in the bias formula to explore the potential consequences of using the RE approach. We demonstrate this process through our application in Section 5.

### 3.1 Connection with Clark and Linzer [15]

In their empirical analyses, Clark and Linzer [15] considered only a special case of the model (1) with $\sigma_{\epsilon }=\sigma_{\alpha }=\sigma_{\gamma }=1$. In this setting, the magnitude of the bias for the RE estimate of β is approximated by $\rho /(1+n\sigma_{X}^{2})$ from Proposition 1. In this case, the bias decreases rapidly as n increases, which reflects their findings that the bias is of little practical importance unless n is small. However, this finding has been interpreted much more broadly in papers referencing [15]. For example, see Dieleman and Templin [24], Gomes [25], and Hopkins et al. [26].

In the original simulation setting of Clark and Linzer [15], the signal‐to‐noise ratio is very large. For example, the inter‐unit reliability (IUR), defined as $R^{2}=\sigma_{\alpha }^{2}/(\sigma_{\alpha }^{2}+\sigma_{\epsilon }^{2}/n)$, is given by n/(n+1), which quickly approaches 1 as n increases. This is unrealistically large for most cluster data applications with medical or social outcomes where, even in relatively large samples, the IUR is typically less than 80% [27, 28, 29].

### 3.2 Connection with Kalbfleisch and Wolfe [9]

Kalbfleisch and Wolfe [9] considered a simpler underlying model for (1) in which the $X_{ij}$ given $\alpha_{i}$ are independent $𝒩(\rho^{*}\frac{\sigma_{X}^{*}}{\sigma_{\alpha }}\alpha_{i},(1-\rho^{*2})\sigma_{X}^{*2})$ variables. In this, $\rho^{*}$ is the correlation between $X_{ij}$ and $\alpha_{i}$, $\sigma_{X}^{2}=(1-\rho^{*2})\sigma_{X}^{*2}$ is the conditional variance of $X_{ij}$ given $\alpha_{i}$, and $\sigma_{X}^{*2}$ is the marginal variance of $X_{ij}$. This model is a special case of TDM (see (8) and (9)) with ρ=1 so that $\gamma_{i}=\frac{\sigma_{\gamma }}{\sigma_{\alpha }}\alpha_{i}$. By Proposition 1, the asymptotic bias (m→∞) of the RE estimate of β under this model is given by the expression (10) with ρ=1.

Kalbfleisch and Wolfe [9] chose $\sigma_{\epsilon }=4$, $\sigma_{\alpha }=1$ and $\sigma_{\gamma }^{2}+\sigma_{X}^{2}=0.09$ with an average sample size n=54. For these parameters, the IUR was approximately 0.77, which is a more realistic value in practice. Thus, these simulations gave substantially larger biases of the RE estimates than did the choices (e.g., $\sigma_{\epsilon }=1$ and $\sigma_{\gamma }^{2}+\sigma_{X}^{2}=1.2$ or 2) in Clark and Linzer [15]. These results help explain the differences between the two papers and show that the RE estimator can have nontrivial bias under realistic scenarios.

## 4 Simulation Studies

In a series of simulation experiments, we assessed the properties of estimation based on FE and RE models over a wide range of parameter settings that varied the signal‐to‐noise ratio $n\sigma_{\alpha }^{2}/\sigma_{\epsilon }^{2}$, the true effect size β, the number of clusters m, the variance of the covariate $\sigma_{X}^{2}$, the variance of the underlying within‐cluster mean of the covariate $\sigma_{\gamma }^{2}$, and the correlation between $\alpha_{i}$ and $\gamma_{i}$ (represented as ρ).

The observations were simulated within the clusters as follows: We first generated m realizations of $\left(\alpha_{i},\gamma_{i}\right)$ from the bivariate normal distribution in (8). For each i=1,…,m, a random sample of n observations of $X_{ij},j=1,\ldots ,n$ was generated from a $𝒩(\gamma_{i},\sigma_{X}^{2})$ distribution and of $\epsilon_{ij},j=1,\ldots ,n$ from a $𝒩(0,\sigma_{\epsilon }^{2})$ distribution. Without loss of generality, we set the population mean μ to 0. Consequently, $Y_{ij}$ was generated from TDM as defined in (8) and (9). This sample also satisfies

(13) $$\begin{array}{l} Y_{ij}=\alpha_{i}+X_{ij}\beta +\epsilon_{ij}. \end{array}$$

In the following subsections, we present results for empirical bias and root mean square error (RMSE) when estimating $\alpha_{i}$ and β based on the FE and RE models for (13). The empirical results were derived from 50,000 replicated data sets in each setting.

### 4.1 Evaluation of β Estimators

#### 4.1.1 Results Depending on the Signal‐to‐Noise ratio $n\sigma_{\alpha }^{2}/\sigma_{\epsilon }^{2}$

We set the number of clusters at m=40, with each cluster having n=50 observations. In addition, we set $\sigma_{\alpha }^{2}=1$, $\sigma_{\gamma }^{2}=0.5$, $\sigma_{X}^{2}=0.5$ and β=1. The correlation ρ varied over the range of −0.9 to 0.9. We specified $\sigma_{\epsilon }^{2}/n$ as 0.08, 0.5, and 1. The corresponding signal‐to‐noise ratio of ${\bar{Y}}_{i}$ took the values 12.5, 2, and 1.

**FIGURE 1:**
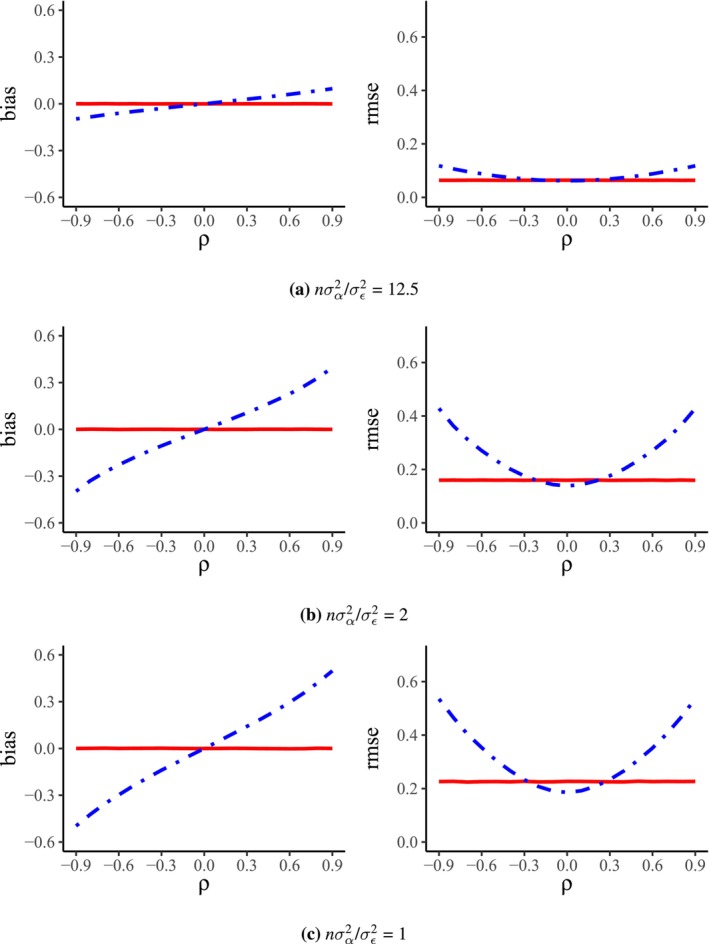
The empirical bias and RMSE (Root mean squared error) for β estimation as they depend on the signal‐to‐noise ratio $n\sigma_{\alpha }^{2}/\sigma_{\epsilon }^{2}$, comparing the fixed effect model (red solid curves) and random effect model (blue dashed curves). We specified $\sigma_{\alpha }^{2}=1$ and $\sigma_{\epsilon }^{2}/n$ as 0.08, 0.5, and 1. The corresponding signal‐to‐noise ratio of ${\bar{Y}}_{i}$, $n\sigma_{\alpha }^{2}/\sigma_{\epsilon }^{2}$, takes the value 12.5, 2 and 1.

Figure 1 shows that the FE estimator of β remains unbiased for all parameter settings, whereas the bias of the RE estimator increases as the signal‐to‐noise decreases. The empirical bias in the RE estimator is very close to that predicted by the asymptotic bias formula (10) in Proposition 1. When ρ is close to 0, the RMSE of the RE estimator is smaller than that of FE, but as |ρ| increases, the RMSE of the RE estimator increases and, in every case, exceeds the RMSE of the FE estimator when |ρ|>0.3.

#### 4.1.2 Results Depending on β

We examined whether variations in the true β impact the performances of the FE and RE models. The simulation settings employed here are consistent with those described in Section 4.1.1, with the exception that $\sigma_{\epsilon }^{2}$ was fixed at 16 while the true β took values 0, 1 and 2.

**FIGURE 2:**
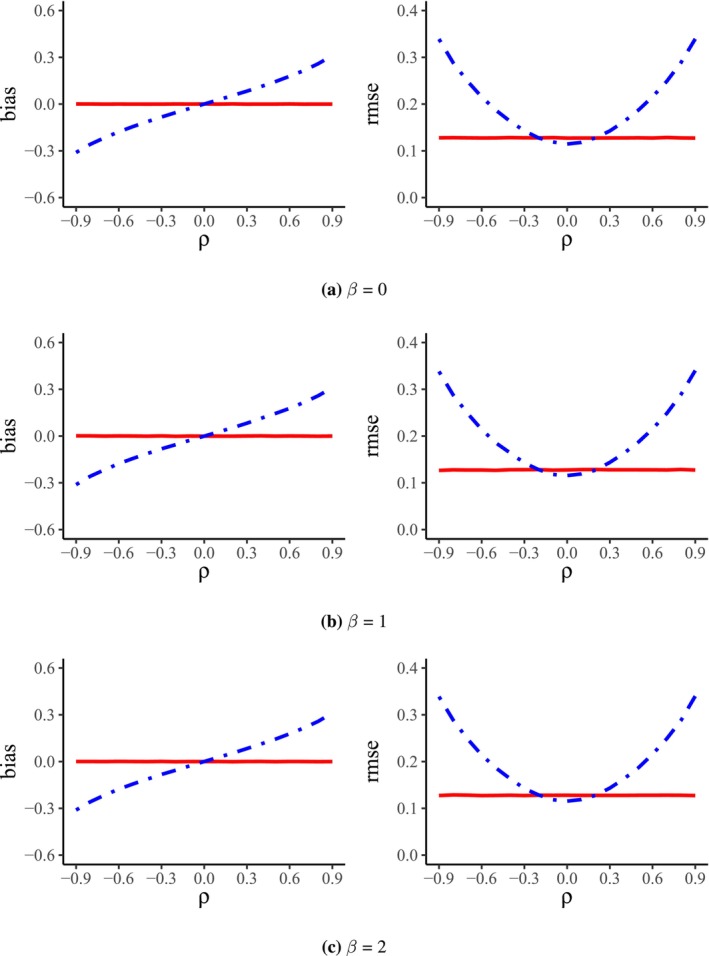
Simulation results of empirical bias and RMSE (root mean square error) in estimating β, for various values of ρ, comparing the fixed effect model (red solid curves) and random effect model (blue dashed curves). The true size of β is varied, with the value from top to bottom being β=0,1,2, respectively.

Figure 2 illustrates that the bias and RMSE of both the FE and RE models are unaffected by changes in β. As expected, the FE approach has nearly unbiased estimation across all settings whereas the RE approach exhibits the same bias for all values of β, although the relative bias is more pronounced when the magnitude of β is small. This matches the lack of β in the asymptotic expression (10). A proof that the bias and RMSE of ${\hat{\beta }}_{\text{RE}}$ do not depend on the value of β is given in the Supporting Information.

#### 4.1.3 Results Depending on the Number of Clusters, m

**FIGURE 3:**
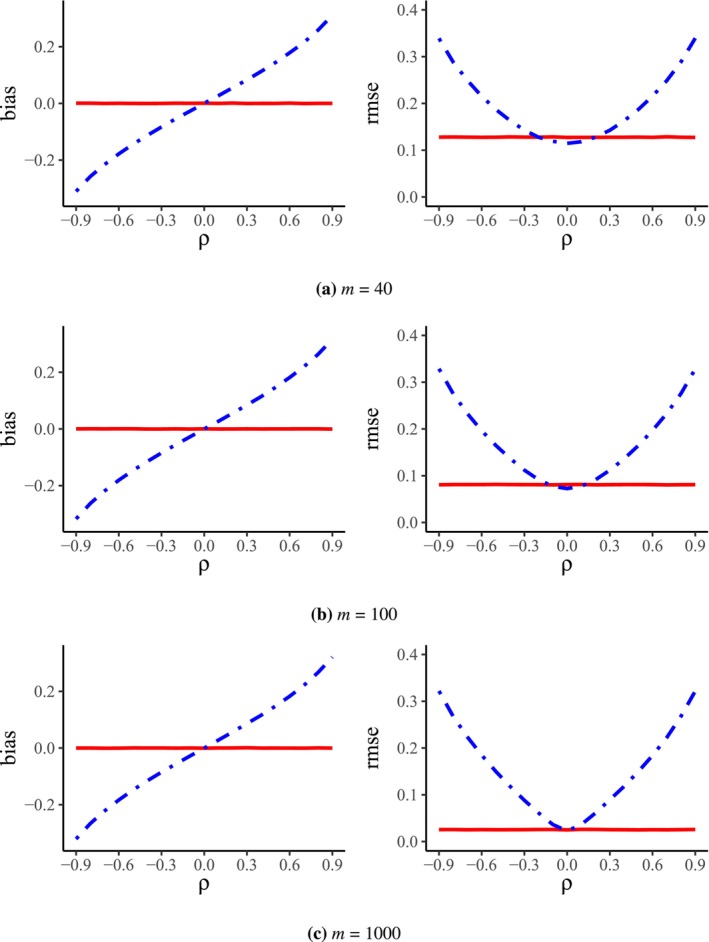
Simulation results display the empirical bias and RMSE (root mean square error) in estimating β, for various values of ρ, comparing the fixed effect model (red solid curves) and the random effect model (blue dashed curves). The number of clusters presented, from top to bottom, is m=40,100, and 1000, respectively.

We conducted a comparison of the bias and RMSE between the FE and RE estimators across different numbers of clusters, specifically m=40, 100, and 1000. The settings for the remaining parameters align with those outlined in Section 4.1.1, but with $\sigma_{\epsilon }^{2}$ being fixed at 16. Figure 3 illustrates that as the number of clusters increases, the bias inherent in the RE estimator persists. Additionally, the superiority of the FE estimator over the RE estimator in terms of RMSE becomes more pronounced.

#### 4.1.4 Results Depending on $\sigma_{X}^{2}/\sigma_{\gamma }^{2}$

**FIGURE 4:**
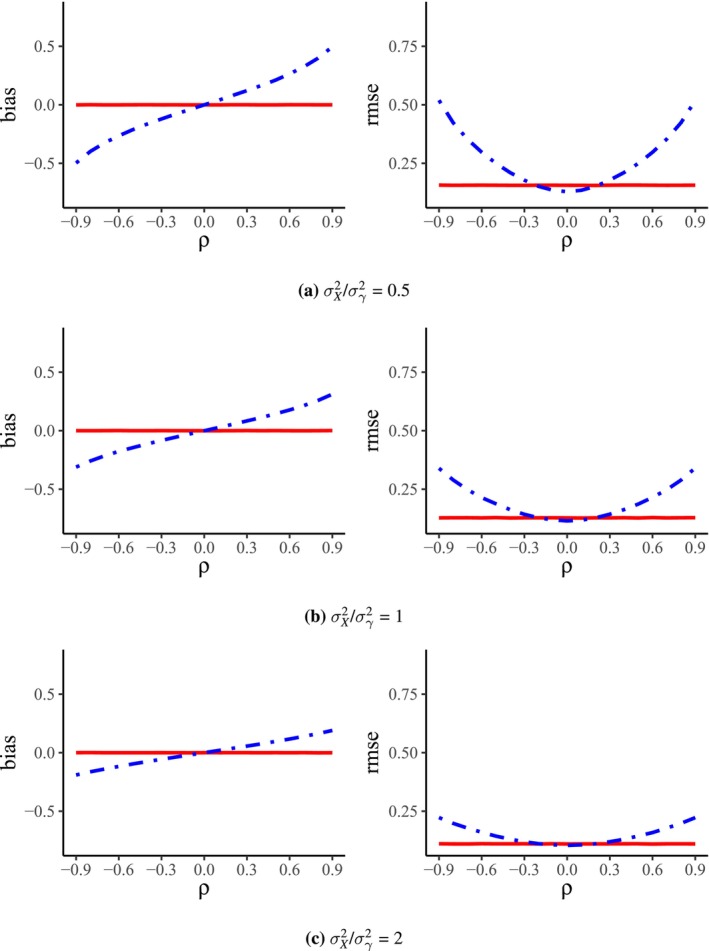
Simulation results showcasing the empirical bias and RMSE (root mean square error) in estimating β, comparing the fixed effect model (red solid line) and random effect model (blue broken line) are presented. The ratios, displayed from top to bottom, are $\sigma_{X}^{2}/\sigma_{\gamma }^{2}=0.5,1$ and 2, respectively, where $\sigma_{X}^{2}+\sigma_{\gamma }^{2}=1$.

Subject to the constraint $\sigma_{X}^{2}+\sigma_{\gamma }^{2}=1$, we investigated the influence of the ratio $\sigma_{X}^{2}/\sigma_{\gamma }^{2}$, on the bias and RMSE of the FE and RE models. Figure 4 illustrates that as the ratio between $\sigma_{X}^{2}$ and $\sigma_{\gamma }^{2}$ decreases (i.e., when the greater variation inherent with covariate $X_{ij}$ is between clusters instead of within clusters), the bias and RMSE of the RE model increase. In other words, the FE model demonstrates superior relative performance as the ratio diminishes. The simulation settings in this subsection generally adhere to those outlined in Section 4.1.1, with $\sigma_{\epsilon }^{2}=16$.

#### 4.1.5 Robustness to Additional Settings

To demonstrate the broader practical relevance of our findings beyond the baseline simulation design, we conducted additional simulation experiments that relax several assumptions of the default setup. Specifically, we examined: (i) heavy‐tailed outcome distributions using scaled Student's t errors with degrees of freedom ν=3,5,30; (ii) binary rather than continuous predictors with marginal prevalence $\pi_{x}\in \{0.3,0.5,0.7\}$; (iii) more complex random effect structures by fixing the first cluster effect $\alpha_{1}$ at prespecified values while estimating β; (iv) unbalanced cluster sizes drawn from a log‐normal distribution with varying degrees of imbalance; (v) multiple endogenous covariates with different levels of inter‐covariate correlation; and (vi) settings where FE and RE lead to substantively different scientific conclusions about the covariate effects. Across all of these settings, the qualitative conclusions remain unchanged: the FE estimator of β is approximately unbiased regardless of ρ, whereas the RE estimator exhibits bias that increases with |ρ|. Full details and results are presented in Supporting Information: Appendix F (Figures S4–S9).

### 4.2 Properties of α Estimators

To compare the FE and RE models in estimating the effect of the cluster, a slightly different simulation process was used for the data within the first cluster. We assumed that cluster 1 and the corresponding $\alpha_{1}$ are of interest. Consequently, we set $\alpha_{1}$ to values of 0, 1.5, and 3, and then simulate $\gamma_{1}$ from the conditional distribution of $\gamma_{1}|\alpha_{1}∼𝒩\left(\rho \sigma_{\gamma }\alpha_{1}/\sigma_{\alpha },(1-\rho^{2})\sigma_{\gamma }^{2}\right)$. Observations for the remaining clusters were generated using the default simulation procedure described previously. All required parameter values remained the same as in Section 4.1.1, with $\sigma_{\epsilon }^{2}=16$. The empirical results reported were based on estimates of $\alpha_{1}$.

**FIGURE 5:**
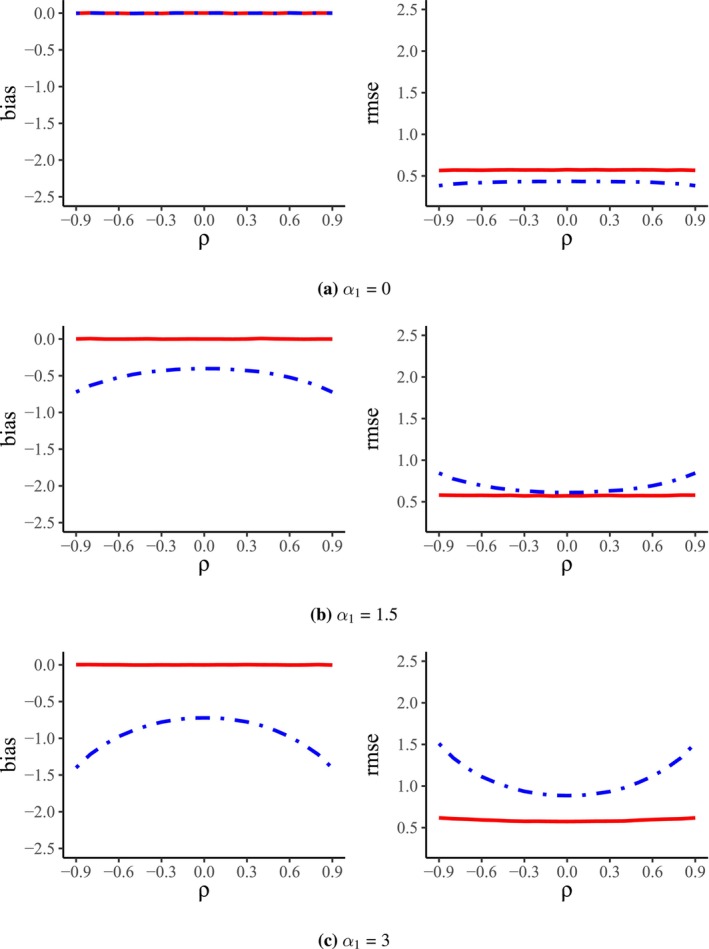
Simulation results illustrate the empirical bias and RMSE (root mean square error) in estimating α, comparing the fixed effect model (red) and the random effect model (blue). The first cluster effect size, presented from top to bottom, is $\alpha_{1}=0,1.5,3$.

As expected, the results depicted in Figure 5 reveal that the FE estimators are approximately unbiased whereas the RE estimators are biased toward zero. Furthermore, the RMSE of the FE estimator is not related to the effect size, whereas the RMSE of the RE estimator increases with the true effect size. When $\alpha_{1}$ is close to 0, the RMSE of the RE estimator is smaller than that of the FE estimator, but for larger $\alpha_{1}$, the reverse is true.

### 4.3 Simulations on the Impact of Estimating η

In this subsection, we used a series of simulations to verify the bias formula in Proposition 1 and assessed the impact of estimating η. Specifically, we calculated the empirical bias based on the estimated coefficients from the simulated data and compared it with the value from the theoretical bias formula. The data were generated similarly to the previous subsections with 100 clusters and 100 observations per cluster. We further assigned $\sigma_{\epsilon }^{2}=1,\sigma_{\alpha }^{2}=1,\sigma_{\gamma }^{2}=0.5$, and $\sigma_{X}^{2}=0.5$ as default. For each of the sensitivity analyses, we changed the values of $\sigma_{\epsilon }^{2},\sigma_{\alpha }^{2}$, and $\sigma_{\gamma }^{2}$, respectively, and compared the differences between the empirical bias and the theoretical bias under different settings. The empirical results were based on 10,000 repetitions. As shown in Figures S1–S3 in the Supporting Information, we conducted sensitivity analyses by varying $\sigma_{\alpha }^{2}$, $\sigma_{\epsilon }^{2}$, and $\sigma_{\gamma }^{2}$. When $\sigma_{\alpha }^{2}$ was relatively large compared with $\sigma_{\epsilon }^{2}$ and $\sigma_{\gamma }^{2}$, the empirical bias closely matched the theoretical bias across values of ρ. In contrast, when both $\sigma_{\epsilon }^{2}$ and $\sigma_{\gamma }^{2}$ were large relative to $\sigma_{\alpha }^{2}$, the discrepancy between empirical and theoretical bias increased. This discrepancy may be due to underestimation of $\sigma_{\alpha }^{2}$ in the fitted random‐effects model, which can produce a larger plug‐in empirical value than the theoretical value of λ(η) based on the true variance components.

## 5 Application

We present two real‐data applications to demonstrate the practical consequences of the choice between Fixed Effects (FE), Random Effects (RE), and Correlated Random Effects (CRE) modeling frameworks. We include CRE models in both applications to illustrate the between‐within decomposition in practice and to show how the choice and completeness of the cluster‐level mean structure affects both coefficient estimation and predicted cluster effects. The first application uses a publicly available educational dataset to illustrate the decomposition of within‐cluster and between‐cluster effects and to show patterns consistent with the analytic derivation derived in Section 3. The second application uses national transplant registry data to demonstrate that these coefficient differences propagate into substantively divergent provider‐level profiling decisions in a high‐dimensional clinical setting.

### 5.1 Application 1: Math Achievement in Schools

We analyzed data from the Early Childhood Longitudinal Study [30], a nationally representative study that tracked more than 18,000 children from kindergarten through fifth grade. We focused on the fifth‐grade cross‐sectional data, using each student's mathematics assessment score as the primary outcome. This score consolidates 18 content areas, including number sense, measurement, geometry, data analysis, and algebra, into a single measure of conceptual knowledge, procedural knowledge, and problem‐solving proficiency. The main predictor of interest was a composite socioeconomic status (SES) measure derived from household characteristics. In addition, the models included two teacher‐reported measures: student‐teacher closeness (Closeness) and the frequency of positive learning behaviors (Learning).

After restricting to complete cases and retaining only schools with more than 10 student records, a screening step that ensures sufficient within‐school information for stable estimation of both FE and RE school effects, the analytical sample comprised 5130 students nested within 370 schools. The baseline model takes the form

$$\begin{array}{ll} {\text{Math Score}}_{ij} & =\mu +\alpha_{i}+\beta_{1}\cdot {\text{SES}}_{ij}+\beta_{2}\cdot {\text{Learning}}_{ij}\\ & \;+\beta_{3}\cdot {\text{Closeness}}_{ij}+\epsilon_{ij}, \end{array}$$

where ij indexes student j within school i, $\alpha_{i}$ represents the school‐specific effect, and $\epsilon_{ij}$ is the residual error. Under the RE specification, the $\alpha_{i}$ are assumed to be independent $𝒩(0,\sigma_{\alpha }^{2})$ variates uncorrelated with the student‐level predictors; the FE specification treats them as fixed unknown parameters.

We began by estimating a baseline Pooled Ordinary Least Squares (OLS) model, which ignores school‐level clustering but provides an initial look at unadjusted associations. To control for unobserved school‐level heterogeneity, we then estimated a standard FE model, followed by an RE model. Because standard RE models assume that student‐level predictors are uncorrelated with the unobserved school‐level random intercepts, an assumption frequently violated in educational and observational data, we transitioned to a CRE framework. To examine how progressively accounting for school‐level composition affects the estimation of student‐level coefficients, we specified three CRE models that sequentially incorporate school‐level aggregate means, each adding one additional contextual covariate. This progressive design allows us to track, one variable at a time, how much of the RE–FE discrepancy is attributable to each source of between‐school confounding. Specifically:

- CRE 1: baseline covariates + school‐level mean of SES;
- CRE 2: CRE 1 + school‐level mean of Learning;
- CRE 3: CRE 2 + school‐level mean of Closeness.

Table 1 reports the estimated regression coefficients across all model specifications. The results provide a direct empirical confirmation of the analytic derivations in Section 3. The coefficient for student‐level SES in the fully specified CRE 3 model (0.157) exactly recovers the FE estimate (0.157), confirming that the CRE framework successfully isolates the unconfounded within‐school effect. By contrast, the standard RE model yields a substantially inflated estimate of 0.183, reflecting the bias that arises from the unmodeled correlation between school‐level SES composition and unobserved school quality ($\hat{\rho }\approx 0.19$). A similar pattern holds for the Learning covariate, whose CRE 3 estimate (0.120) aligns with the FE estimate, whereas the RE estimate (0.117) is slightly attenuated. The Closeness covariate is not statistically significant under any specification.

**TABLE 1:** Estimated regression coefficients for models predicting student math scores (n=5130 students nested within 370 schools with more than 10 records each). The table compares Pooled Ordinary Least Squares (OLS), Fixed Effects (FE), Random Effects (RE), and three specifications of Correlated Random Effects (CRE) models. Student‐level predictors include student socioeconomic status (SES), teacher‐reported Learning, and Closeness. CRE models 1–3 progressively incorporate the school‐level aggregate means of these predictors to decompose effects into within‐school and between‐school components. Consistent with the analytic derivations in Section 3, the within‐school effect of SES in the fully specified CRE 3 model (0.157) exactly recovers the FE estimate, eliminating the bias present in the standard RE model (0.183). Standard errors are reported in parentheses.

Variable	Pooled	FE	RE	CRE 1	CRE 2	CRE 3
*Student‐level Predictors*						
SES	0.201***	0.157***	0.183***	0.157***	0.157***	0.157***
	(0.007)	(0.009)	(0.008)	(0.009)	(0.009)	(0.009)
Learning	0.115***	0.120***	0.117***	0.117***	0.119***	0.120***
	(0.006)	(0.007)	(0.006)	(0.006)	(0.007)	(0.007)
Closeness	0.003	−0.000	0.001	0.002	0.002	−0.000
	(0.006)	(0.006)	(0.006)	(0.006)	(0.006)	(0.006)
*School‐level Predictors*						
Mean SES				0.111***	0.114***	0.114***
				(0.017)	(0.018)	(0.018)
Mean Learning					−0.030	−0.034
					(0.027)	(0.027)
Mean Closeness						0.018
						(0.016)
Intercept	1.313***		1.315***	1.302***	1.390***	1.338***
	(0.028)		(0.029)	(0.029)	(0.084)	(0.096)

*Note:* Standard errors are in parentheses. FE = Fixed Effects; RE = Random Effects; CRE = Correlated Random Effects. *** p<0.001, ** p<0.01, * p<0.05.

**FIGURE 6:**
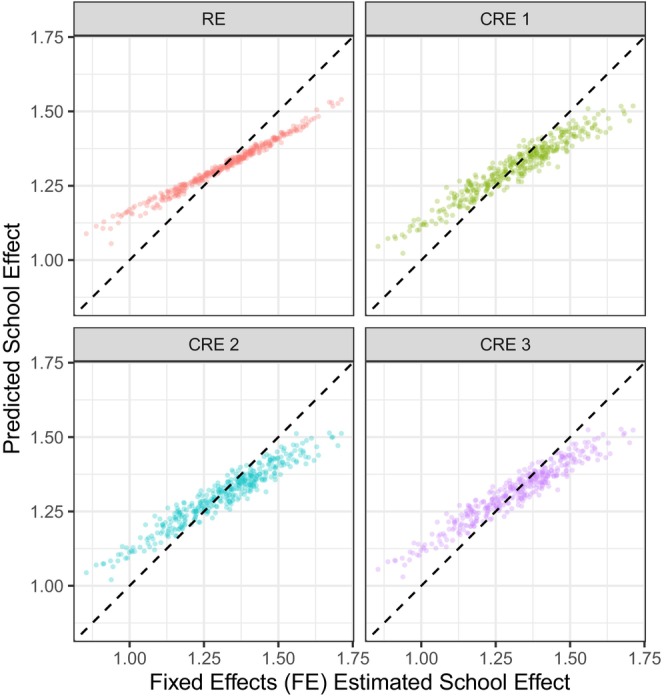
Scatter plots comparing Fixed Effects (FE) estimated school effects (x‐axis) against predicted school effects (y‐axis) derived from a standard Random Effects (RE) model and three progressively specified Correlated Random Effects (CRE) models, for the 370 schools with more than 10 student records (5130 students total). The dashed black line represents a 1:1 reference line where predictions perfectly match the FE estimates. Across all mixed‐effects specifications, the predicted school effects exhibit shrinkage toward the global intercept, though the CRE models track the FE baseline more closely than the standard RE model.

To explore the implications for school‐level inference, Figure 6 plots the predicted school effects from the RE and CRE models against the unconstrained FE estimates for the 370 retained schools. While all mixed‐effects specifications exhibit shrinkage toward the global mean relative to the FE estimates, the predicted effects from the CRE models align with the FE baseline more closely than those from the standard RE model, consistent with the coefficient‐level correction documented in Table 1. Nevertheless, the CRE predictions remain more tightly concentrated than the FE estimates, confirming that while the CRE framework corrects bias in the regression coefficients, the predicted cluster effects retain the variance‐reducing properties inherent to random‐effects estimation.

### 5.2 Application 2: Kidney Transplant Center Profiling

To demonstrate the broader clinical relevance of these findings in a large‐scale setting with more predictors, we conducted an additional analysis using data from the Organ Procurement and Transplantation Network (OPTN). The outcome of interest is the patient's Estimated Glomerular Filtration Rate (eGFR) at hospital discharge, a standard clinical measure of early renal function and transplant success. The initial cohort includes all 28,151 adult patients (age ≥18) who received a kidney transplant between July 2019 and June 2021 across 207 transplant centers. To ensure stable estimation of center‐level effects, we restricted the analysis to centers with at least 20 patients, yielding a final analytical sample of 27,891 patients nested within 179 centers. The risk‐adjustment models incorporate 68 patient‐level clinical and demographic covariates, including the critical baseline measure of pretransplant eGFR.

We evaluated three primary modeling frameworks: FE, RE, and CRE. As in the educational application, we are interested in how different levels of adjustment at the center level affect the estimation of patient‐level coefficients. Because this clinical dataset involves 68 covariates rather than three, it also provides an opportunity to examine how the number and selection of center‐level contextual means govern the rate at which the CRE estimates converge to the FE baseline. To further separate within‐center and between‐center components, we utilized a progressive CRE modeling strategy. Building upon the baseline patient‐level covariates, we sequentially introduced center‐level aggregate means to explicitly model contextual effects. Specifically, the three CRE models are defined as follows:

- CRE I: baseline covariates + center‐level mean of donor eGFR;
- CRE II: baseline covariates + center‐level mean of donor eGFR + 15 additional center‐level covariate means;
- CRE III: baseline covariates + center‐level means of all 68 patient‐level characteristics.

The sequence from CRE I to CRE III is intended to illustrate the role of increasingly rich contextual adjustment, not to imply that a fully saturated CRE model is automatically the preferred practical model. In a high‐dimensional registry analysis with 68 patient‐level covariates and 179 centers, selecting the relevant center‐level means is a substantive and statistical modeling problem. Including too few means may leave residual bias, whereas including all means may create collinearity and reduce interpretability. This illustrates an important practical distinction between FE and CRE: FE estimates the within‐center coefficient without selecting between‐center predictors, whereas CRE requires an explicit specification of the cluster‐level mean structure.

**FIGURE 7:**
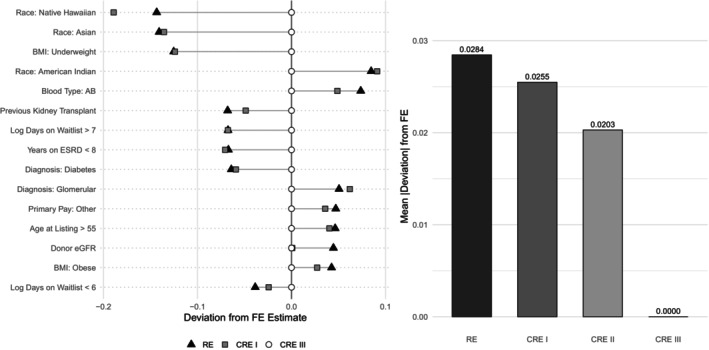
Deviation of patient‐level risk‐adjustment coefficients from the Fixed Effects (FE) baseline across Random Effects (RE) and Correlated Random Effects (CRE) models for transplant center profiling using OPTN data (N=27,891 patients; 179 centers). **Left panel:** For the 15 covariates with the largest absolute RE‐to‐FE discrepancy, each point shows the signed deviation of a given model's coefficient estimate from the corresponding FE estimate; the vertical line at zero represents the FE reference. Gray segments connect zero to the RE point, encoding the bias magnitude. Filled black triangles denote RE, gray squares denote CRE I (adjusting for center‐mean donor eGFR only), and open circles denote CRE III (adjusting for all 68 center‐level covariate means). The convergence of CRE III to zero shows that the fully specified CRE model matches the FE within‐center coefficient estimates in this application. Right panel: Mean absolute deviation from FE across all 68 covariates for each model. The deviation decreases monotonically from RE to CRE I to CRE II to CRE III, illustrating progressive elimination of bias as center‐level contextual means are incorporated.

Figure 7 compares the patient‐level risk‐adjustment coefficient estimates across the FE, RE, and CRE frameworks. The left panel displays the deviation of each model's coefficient from the unconstrained FE baseline for the 15 covariates exhibiting the largest discrepancies between the RE and FE estimates. The gray segments represent the magnitude of the RE‐FE discrepancy for each covariate, while the CRE I and CRE III estimates are shown as separate points. Consistent with our findings in the educational dataset, the CRE estimates cluster near zero deviation, confirming that the CRE framework recovers the unconfounded within‐center coefficients. By contrast, the RE model introduces notable structural deviation from the FE benchmark across a range of clinically important covariates. The right panel quantifies this pattern in aggregate: the mean absolute deviation from FE decreases monotonically from RE through CRE I and CRE II to CRE III, where it reaches zero by construction. These results show patterns consistent with the theoretical bias mechanism documented in Section 3.

While the primary focus of this paper is on the consistent estimation of the regression coefficients β, the coefficient‐level bias documented above has direct downstream consequences for provider profiling. To illustrate this, we compared the predicted center effects across all five modeling frameworks.

**FIGURE 8:**
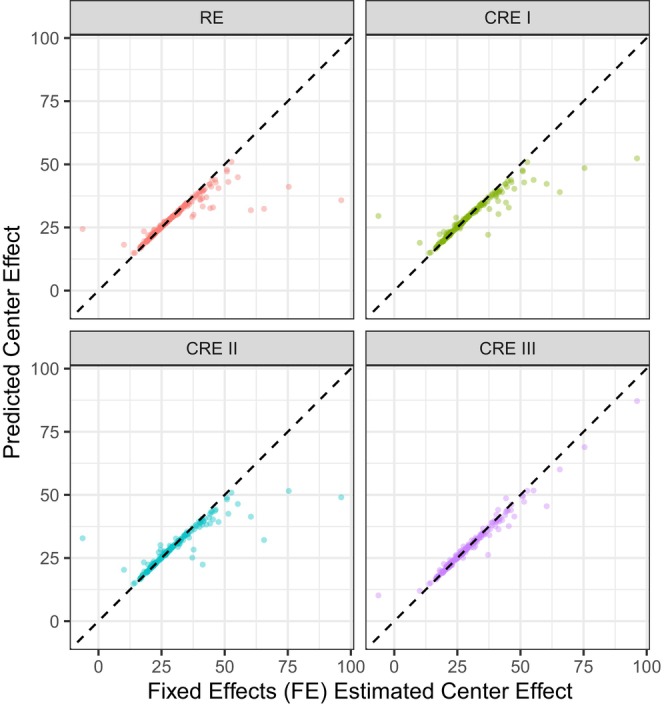
Scatter plots comparing the unconstrained Fixed Effects (FE) estimated transplant center effects (x‐axis) against the predicted center effects (y‐axis) derived from a standard Random Effects (RE) model and three progressively specified Correlated Random Effects (CRE) models, using OPTN kidney transplant data (N=27,891 patients; 179 centers with ≥20 patients). All center effects reflect predicted discharge eGFR; the dashed black line represents a 1:1 reference line indicating perfect alignment with the FE estimates. The models progress from standard RE to CRE I (adjusting for center‐mean donor eGFR), CRE II (adding 15 center‐level covariate means), and CRE III (incorporating center‐means for all 68 patient‐level characteristics). Under the standard RE model, the predicted center effects deviate from the FE baseline, reflecting a combination of empirical Bayes shrinkage and structural bias. As center‐level aggregates are progressively incorporated into the CRE framework, the predicted provider effects align more closely with the FE estimates, confirming that the coefficient‐level bias documented in Figure 7 propagates into the predicted center effects.

Figure 8 plots the predicted center effects from the RE and three CRE models against the unconstrained FE estimates. Under the standard RE specification, the predicted center effects deviate substantially from the 45‐degree reference line, reflecting a combination of empirical Bayes shrinkage and structural bias induced by the unmodeled correlation between patient‐level severity and unobserved center effects. As center‐level means are sequentially introduced into the CRE framework, the predictions progressively align with the FE baseline. Under the fully specified CRE III, the predicted center effects closely align with the FE estimates, confirming that the CRE framework accounts for the modeled between‐center mean structure. These patterns mirror the coefficient‐level convergence documented in Figure 7 and provide visual confirmation that the bias in β propagates directly into the predicted provider effects.

To further examine the practical consequences of these prediction differences, we translated the predicted center effects into regulatory outlier classifications and compared the flagging outcomes across models. The full results, including cross‐tabulations of flagging agreement, are reported in Supporting Information: Appendix G. In summary, up to 68 of the 179 centers (38.0%) receive a different regulatory designation depending on the choice of model, reinforcing the practical message of this paper: the bias in β is not merely a theoretical concern but propagates into substantively different provider‐level decisions. These flagging comparisons should be interpreted as sensitivity analyses rather than as evidence that any one set of classifications is definitively correct. The correct operational classification depends on choices that go beyond coefficient estimation, including the null hypothesis for provider comparison, the desired balance between Type I and Type II error, the role of shrinkage, and the clinical or regulatory objectives of the profiling program. Our point is that the bias in β and the resulting differences in predicted center effects can materially change downstream classifications, so the modeling choice should be made explicitly rather than treated as a minor technical detail. A rigorous treatment of the testing and flagging problem, including the appropriate choice of null hypothesis and the control of error rates, is the subject of ongoing work and is beyond the scope of this paper.

## 6 Discussion

The choice between FE and RE models in clustered data analysis depends on various factors, including the type of question to be addressed. If one is interested in simultaneously estimating all the cluster effects, an argument can be made for incorporating likely connections between the cluster effects through the use of an RE approach. If the outcome of interest is the evaluation of individual clusters, for example in the problems of profiling healthcare providers, the FE method is better suited and provides a much more powerful approach to identifying extreme values. See, for example, Kalbfleisch and Wolfe [9]; Xia et al. [31]. In effect, RE methods, when appropriate, reduce the overall rate of Type I errors while also increasing the rate of Type II errors. This is appropriate if avoiding false positives is the dominant priority, but with profiling healthcare providers, the greater risk is not identifying providers whose service is well outside the range of acceptability.

An often unstated but implied assumption for a simple random‐intercepts model for clustered data is that the covariates in the model are independent of the random effects. In the provider profiling example, this means that the types of patients who receive service from a given provider do not depend on the provider's cluster effect. This is violated, for example, if sicker patients tend to go to hospitals with higher (better) random effects. Many authors do not seem to recognize that this RE condition is required, whereas others have acknowledged the issue but assume that it is of little practical importance and can be safely ignored. We have attempted to give a clear exposition of the size of possible biases in the random‐effects estimation of the regression parameters when the independence assumption is not satisfied. This model includes the simulation designs of both Kalbfleisch and Wolfe [9] and Clark and Linzer [15] as special cases, and provides a framework for reconciling the differing conclusions presented in those studies. We have also developed a general model that allows for the dependence between the covariates and the cluster effects and obtained an asymptotic formula for the bias in the estimation of β using the usual RE analysis when the signal‐to‐noise ratio is known. The accuracy of this theoretical formula has been confirmed through simulation experiments as described in this report. When the covariates are dependent on the cluster effects, the RE estimation of the cluster effects is also affected, but the shrinkage estimators that arise from that model already exhibit substantial biases even when the covariates are independent of the cluster effects.

Although the existence of bias under correlated random effects is well known, its practical magnitude remains an important applied question. The paper by Clark and Linzer [15] has been widely cited as evidence that RE bias is often negligible, whereas Kalbfleisch and Wolfe [9] found much larger discrepancies in provider‐profiling settings. Our purpose is not to reestablish that bias can occur, but to explain analytically why these numerical conclusions differ and to identify the realistic design features under which the bias can affect scientific and policy conclusions. In this sense, the simulations complement the theory by translating the bias formula into coverage, MSE, Type I error, and provider‐classification consequences under interpretable study designs.

It is worth noting that the bias in estimating β in the RE model can be addressed in various ways. It is possible, for example, to include both between and within comparisons for covariates in the model and, as discussed in Neuhaus and Kalbfleisch [16] and Neuhaus and McCulloch [19], this will allow consistent estimation of both the between and within dependence of the outcomes on the covariates when the relevant decomposition is correctly specified. This CRE/BW approach is an important alternative to the standard RE model and is the motivation for the progressive CRE analyses in Section 5. However, the correction is not automatic: it requires the analyst to include the relevant cluster‐level means of covariates associated with the cluster effect. In high‐dimensional applications, partially specified CRE models can leave residual bias, and the residual bias may depend on which contextual means are selected. The FE estimator avoids this specification choice for the within‐cluster coefficient β, although it does not provide the same shrinkage interpretation for cluster‐effect prediction. An alternative approach is to use fixed effects to estimate the effect within clusters of the regression parameter β, and then to use random effects to estimate the effects between clusters.

While our primary focus is on the estimation of population‐level regression coefficients, we acknowledge that different priorities arise when the objective is the evaluation of individual clusters (e.g., healthcare provider profiling). In contexts where identifying extreme values is the primary goal, FE methods are often better suited, as they provide a more direct approach to identifying outliers without the ‘shrinkage’ toward the mean inherent in standard RE models [9, 31]. As noted by McCulloch and Neuhaus [32], standard RE methods are designed to minimize overall mean squared error, which often leads to an increased Type II error rate regarding the detection of extremes. While RE models effectively reduce the global Type I error rate, they may underperform in identifying specific providers whose performance falls well outside the acceptable range—a critical risk in clinical quality oversight.

Finally, we note that the closed‐form expression in (10) is derived under the TDM, a specific Gaussian dependence structure. The formula is useful as a transparent device for understanding how bias depends on signal‐to‐noise ratio, cluster size, and the within‐ versus between‐cluster variation in the covariate, but it should not be interpreted as covering all possible forms of dependence between covariates and cluster effects.

## Author Contributions

All authors contributed to the study's conception and design, and participated in writing and editing the manuscript. Xiangeng Fang and Yubo Shao developed the source code and assisted with data analysis. All authors reviewed and approved the final manuscript.

## Funding

This work was supported by the National Institute of Diabetes and Digestive and Kidney Diseases (Grant No. DK129539) and Health Resources and Services Administration contract HHSH250‐2019‐00001C.

## Disclosure

The authors have nothing to report.

## Conflicts of Interest

The authors declare no conflicts of interest.

## Supporting information




**Data S1.** **Table S1.** Cross‐tabulation of transplant center regulatory flagging status across statistical models (n=179 centers with ≥ 20 patients; OPTN, July 2019‐June 2021; N=27,891 patients). Each 3×3 sub‐panel compares the outlier classification assigned by the row method against the column method, based on discharge eGFR risk‐adjusted for 68 patient‐level covariates. Bold diagonal entries denote concordant classifications. W = Worse, A = Average, B = Better. Models: FE= Fixed Effects, RE = RandomEffects, CRE I = additionally adjusting for center‐mean donor eGFR, CRE II = additionally adjusting for 15 additional center‐level covariate means (16 in total, including donor eGFR), CRE III = additionally adjusting for all 68 center‐level covariate means. **Figure S1.** Comparison between the empirical bias (orange), with estimated η, and theoretical bias (black), assuming η is known, of random effects models inestimating β, as $\sigma_{\alpha }^{2}$ varies from 0.5 to 4 (top to bottom), and $\sigma_{\epsilon }^{2}$ varies from 1 to 8 (left to right). The other parameters are set as follows: number of clusters m=100, cluster size $n=100,\sigma_{X}^{2}=0.5$, and $\sigma_{\gamma }^{2}=0.5$. **Figure S2.** Comparison between the empirical bias (orange), with estimated η, and theoretical bias (black), assuming η is known, of random effects models in estimating β, as $\sigma_{\alpha }^{2}$ varies from 0.5 to 4 (top to bottom), and $\sigma_{\gamma }^{2}$ varies from 0.5 to 4 (left to right). The other parameters are set as follows: number of clusters m=100, cluster size $n=100,\sigma_{X}^{2}=0.5$, and $\sigma_{\epsilon }^{2}=1$. **Figure S3.** Comparison between the empirical bias (orange), with estimated η, and theoretical bias (black), assuming η is known, of random effects models in estimating β, as $\sigma_{2}^{\gamma }$ varies from 0.5 to 4 (top to bottom), and $\sigma_{\epsilon }^{2}$ varies from 1 to 8 (left to right). The other parameters are set as follows: number of clusters m=100, cluster size $n=100,\sigma_{X}^{2}=0.5$, and $\sigma_{\alpha }^{2}=1$. **Figure S4.** Empirical bias and RMSE for estimating β under error distributions with different tail behaviors. Panels correspond to $t_{v}$ errors with v=30, 5, and 3, representing approximately normal, moderately heavy‐tailed, and strongly heavy‐tailed error distributions, respectively. Results are shown across values of ρ, comparing the fixed‐effects model (red solid curves) and the random‐effects model (blue dashed curves). **Figure S5.** Empirical bias and RMSE for estimating β across values of ρ, comparing the fixed effects model (red solid curves) and the random‐effects model (blue dashed curves). Panels correspond to binary predictors with marginal prevalence $\pi_{x}=0.3$, 0.5, and 0.7, respectively. **Figure S6.** Empirical bias and RMSE for estimating β across values of ρ, comparing the fixed effects model (red solid curves) and the random‐effects model (blue dashed curves). Panels cor respond to different values of the first cluster effect $\alpha_{1}=0$, 1.5, and 3, respectively. **Figure S7.** Empirical bias and RMSE for estimating β across values of ρ, comparing the fixed effects model (red solid curves) and the random‐effects model (blue dashed curves). Panels corre spond to different levels of cluster‐size variability with $\sigma_{n}=0.1$, 0.6, and 1.0, representing mild, moderate, and high imbalance, respectively **Figure S8.** Empirical bias and RMSE for estimating β with p=2 endogenous covariates, averaged across the two regression coefficients. Panels correspond to inter‐covariate correlations $\rho_{\gamma }=0$, 0.2, and 0.4. Results are shown across values of ρ, comparingthefixed‐effects model (red solid curves) and the random‐effects model (blue dashed curves). **Figure S9.** Simulation comparing the inferential quality of the Fixed Effects (FE) and Random Effects (RE) estimators of β as a function of the true effect size (m=40, $n_{i}=20$, ρ=0.6, 5000 replications per grid point). **Panel (A):** Empirical coverage of the 95% Wald confidence interval. The FE estimator (solid line, filled circles) maintains coverage at the nominal 95% level (dotted reference line) across all values of β. The RE estimator (dashed line, open circles) falls to 91 93%, indicating systematic undercoverage driven by the constant positive bias. **Panel (B).** Mean squared error decomposed into variance (light shading) and ${\text{bias}}^{2}$ (dark shading). The FE panel contains only variance; the RE panel shows a constant ${\text{bias}}^{2}$ floor that elevates MSE above the FE level despite the RE estimator's lower variance. **Panel (C):** Rejection rate for $H_{0}:\beta =0$ at the 5% level (dotted reference line). At β=0, FE rejects at approximately 5% (correct calibration), whereas RE exhibits inflated Type I error. For positive β, the RE model appears to have higher power, but this apparent advantage is driven by the same bias that inflates Type I error and degrades coverage.

## Data Availability

The data that support the findings of this study are available in User's Manual for the ECLS‐K:2011 Kindergarten Data File and at: https://nces.ed.gov/use‐work/resource‐library/report/users‐manualdata‐file‐documentation/users‐manual‐ecls‐k2011‐kindergarten‐data‐file‐and‐electronic‐codebook‐public‐version.
