# Supplementary material for: Fixed or Random Effects: Analysis of Clustered Data
Source: Stat Med. 2026 Aug 6;45(18-19):e70687. doi: 10.1002/sim.70687 (PMC13445157; doi:10.1002/sim.70687)
Supplement: Supplementary file 1 [file SIM-45-0-s001.pdf]

Appendix for

Fixed or Random Effects: Analysis of Clustered Data

Kevin He, Xiangeng Fang, Yubo Shao, Nicholas Hartman, John D. Kalbfleisch

A Proof of Proposition 1

Consider the linear regression model

$$Y_{ij} = \mu + \alpha_i + X_{ij}\beta + \epsilon_{ij},$$

where X_{ij} is a scalar random variable ($p = 1$), and suppose that the underlying model, TDM specified by (8) and (9) holds. Since $X_{ij}|\gamma_i, \alpha_i \overset{i.i.d}{\sim} \mathcal{N}(\gamma_i, \sigma_X^2)$, we decompose X_{ij} as $X_{ij} = \gamma_i + \omega_{ij}$ with ω_{ij} independently and identically following a distribution $\mathcal{N}(0, \sigma_X^2)$. The joint distribution of X_{ij}, α_i and γ_i is given by,

$$\begin{pmatrix} X_{ij} \\ \alpha_i \\ \gamma_i \end{pmatrix} \sim \mathcal{N}\left(\begin{pmatrix} 0 \\ 0 \\ 0 \end{pmatrix}, \begin{pmatrix} \sigma_\gamma^2 + \sigma_X^2 & \rho\sigma_\alpha\sigma_\gamma & \sigma_\gamma^2 \\ \rho\sigma_\alpha\sigma_\gamma & \sigma_\alpha^2 & \rho\sigma_\alpha\sigma_\gamma \\ \sigma_\gamma^2 & \rho\sigma_\alpha\sigma_\gamma & \sigma_\gamma^2 \end{pmatrix} \right).$$

For $\rho \neq 0$, X_{ij} and X_{ik}, $j \neq k$, are correlated with α_i, and the joint distribution of $\bar{X}_i = \sum_{j=1}^n X_{ij}/n$ and α_i is given by

$$\begin{pmatrix} \bar{X}_i \\ \alpha_i \end{pmatrix} \sim \mathcal{N}\left(\begin{pmatrix} 0 \\ 0 \end{pmatrix}, \begin{pmatrix} \sigma_\gamma^2 + \sigma_X^2/n & \rho\sigma_\alpha\sigma_\gamma \\ \rho\sigma_\alpha\sigma_\gamma & \sigma_\alpha^2 \end{pmatrix} \right).$$

Thus, the conditional distribution of α_i given $\bar{X}_i$ is

$$\alpha_i|\bar{X}_i \sim \mathcal{N}\left(\frac{\rho\sigma_\alpha\sigma_\gamma}{\sigma_\gamma^2 + \sigma_X^2/n}\bar{X}_i, \frac{(1-\rho^2)\sigma_\gamma^2 + \sigma_X^2/n}{\sigma_\gamma^2 + \sigma_X^2/n}\sigma_\alpha^2 \right).$$

Now, TDM can be reformulated as

$$Y_{ij} = \mu + \alpha_i^* + \beta_B\bar{X}_i + \beta_W(X_{ij} - \bar{X}_i) + \epsilon_{ij},$$

where $\beta_W = \beta$,

$$\alpha_i^* \sim \mathcal{N}\left(0, \frac{(1-\rho^2)\sigma_\gamma^2 + \sigma_X^2/n}{\sigma_\gamma^2 + \sigma_X^2/n}\sigma_\alpha^2\right),$$

and

$$\beta_B - \beta_W = \frac{\rho\sigma_\alpha\sigma_\gamma}{\sigma_\gamma^2 + \sigma_X^2/n}.$$

Thus, the TDM can be rewritten as a correlated random effects (CRE) model, which was originally proposed by Mundlak (1978) [1] and also known as the between-within (BW) approach [2], allowing α_i to be correlated with X_{ij} through a particular form. To simplify notation, we assume an equal number of observations within each cluster, which allows us to establish the connection between the TDM and the CRE model.

By Scott and Holt (1982) [3], for known η, the RE estimator is given by

$$\hat{\beta}_{\text{RE}} = \hat{\beta}_W + \lambda(\eta)(\hat{\beta}_B - \hat{\beta}_W),$$

where η is defined in (6), $\lambda(\eta)$ is defined in (7), with

$$\hat{\tau} = \frac{\sum_i n(\bar{X}_i - \bar{X})^2}{\sum_i \sum_j (X_{ij} - \bar{X})^2}. \tag{A1}$$

Moreover, since

$$\begin{pmatrix} X_{ij} \\ \bar{X}_i \end{pmatrix} \sim \mathcal{N}\left(\begin{pmatrix} 0 \\ 0 \end{pmatrix}, \begin{pmatrix} \sigma_\gamma^2 + \sigma_X^2 & \sigma_\gamma^2 + \sigma_X^2/n \\ \sigma_\gamma^2 + \sigma_X^2/n & \sigma_\gamma^2 + \sigma_X^2/n \end{pmatrix} \right), \quad E(\bar{X}_i | X_{ij}) = \frac{\sigma_\gamma^2 + \sigma_X^2/n}{\sigma_\gamma^2 + \sigma_X^2} X_{ij},$$

we have

$$E(\bar{X}_i | X_{ij} = s+1) - E(\bar{X}_i | X_{ij} = s) = \frac{\sigma_\gamma^2 + \sigma_X^2/n}{\sigma_\gamma^2 + \sigma_X^2}.$$

Define $\tau = E(\bar{X}_i | X_{ij} = s+1) - E(\bar{X}_i | X_{ij} = s)$, then $\hat{\tau}$ given in (A1) is an unbiased estimator for τ. It follows that the asymptotic bias of $\hat{\beta}_{\text{RE}}$ is

$$\lambda(\eta)(\beta_B - \beta_W) = \frac{(1-\eta)\rho\sigma_\alpha\sigma_\gamma}{(1-\eta)(\sigma_\gamma^2 + \sigma_X^2) + (n-1)\eta\sigma_X^2}. \tag{A2}$$

Moreover, if η, the true intra-cluster correlation coefficient of $\delta_{ij} = \alpha_i + \epsilon_{ij}$, is known, the asymptotic bias is given by

$$\lambda(\eta)(\beta_B - \beta_W) = \left\{ \frac{\sigma_\gamma^2 + \sigma_X^2}{\rho\sigma_\alpha\sigma_\gamma} + \frac{\sigma_\alpha\sigma_X^2}{\rho\sigma_\gamma\sigma_\epsilon^2/(n-1)} \right\}^{-1} = \frac{\rho\sigma_\alpha\sigma_\gamma}{\sigma_X^2} \left\{ 1 + \frac{\sigma_\gamma^2}{\sigma_X^2} + \frac{(n-1)\sigma_\alpha^2}{\sigma_\epsilon^2} \right\}^{-1}.$$

In practice, η is unknown. As discussed in Shen et al. (2008) [4], the estimate of η under the RE model is not in general a consistent estimator of η. If $\hat{\eta}$ converges to η^*, then the asymptotic bias is $\lambda(\eta^*)(\beta_B - \beta_W)$.

B Proof of Corollary 1

Consider the group of transformations $Y_{ij} \to Y_{ij} + CX_{ij} = Y_{ij}^*, i = 1, \ldots, m; j = 1, \ldots n$ where $-\infty < C < \infty$. This induces a transformation in the parameter space with $\beta_W \to \beta_W + C = \beta_W^*$ and $\beta_B \to \beta_B + C = \beta_B^*$, whereas all other parameters are left invariant. Since, η and $\hat{\eta}$ are left invariant, $\hat{\beta}_{\text{RE}} \to \hat{\beta}_{\text{RE}} + C = \hat{\beta}_{\text{RE}}^*$ and $\beta \to \beta + C = \beta^*$. Thus, the bias and mean squared error of $\hat{\beta}_{\text{RE}}$ are the same as those for $\hat{\beta}_{\text{RE}}^*$ for any C, and so the bias and MSE of $\hat{\beta}_{\text{RE}}$ do not depend on β.

C Proof of Corollary 2

This is straightforward.

D Proof of Corollary 3

a) Consider the linear regression model

$$Y_{ij} = \mu + \alpha_i + X_{ij}\beta + \mathbf{Z}_{ij}^{\top}\theta + \epsilon_{\mathbf{ij}}$$

where $\mathbf{Z}_{ij}$ is a vector of q-dimensional covariates that are jointly independent of α_i although may be dependent on the scalar X_{ij}. As before, suppose that the underlying model is TDM. If $\mathbf{Z}_{ij}$ is independent of X_{ij}, then it is immediate that the estimator of β is unchanged by $\mathbf{Z}_{ij}$ and the bias formula (A2) still applies.

b) If $\mathbf{Z}_{ij}$ and X_{ij} are not independent, then their distribution, conditional on α_i and γ_i, can be written as

$$\begin{pmatrix} X_{ij} \\ \mathbf{Z}_{ij} \end{pmatrix} \sim \mathcal{N}\left(\begin{pmatrix} \gamma_i \\ \boldsymbol{\mu}_{\mathbf{Z}} \end{pmatrix}, \begin{pmatrix} \sigma_X^2 & \boldsymbol{\Sigma}_{X,\mathbf{Z}} \\ \boldsymbol{\Sigma}_{\mathbf{Z},X} & \boldsymbol{\Sigma}_{\mathbf{Z}} \end{pmatrix} \right),$$

where $E(\mathbf{Z}_{ij}|\alpha_i, \gamma_i) = \boldsymbol{\mu}_{\mathbf{Z}}$ is a constant since $\mathbf{Z}_{ij}$ is independent of α_i. Given α_i and γ_i, we assume that X_{ij} and $\mathbf{Z}_{ij}$ are i.i.d, for $j = 1, \ldots, n$. So, for example, that $\mathbf{Z}_{ij}$ is independent of $X_{ij'}$ and $\mathbf{Z}_{ij'}$ for all $j \neq j'$. Note that $E(X_{ij}|\gamma_i, \alpha_i) = \gamma_i$.

Consider the projection of X_{ij} on $\mathbf{Z}_{ij} - \boldsymbol{\mu}_{\mathbf{Z}}$ and let

$$X_{ij}^* = X_{ij} - (\mathbf{Z}_{ij} - \boldsymbol{\mu}_{\mathbf{Z}})^{\top} \boldsymbol{\Sigma}_{\mathbf{Z}}^{-1} \boldsymbol{\Sigma}_{\mathbf{Z},X},$$

so that $\mathbf{Z}_{ij}$ is jointly independent of α_i and X_{ij}^*. The model can then be rewritten as

$$Y_{ij} = \mu^* + \alpha_i + X_{ij}^*\beta + \mathbf{Z}_{ij}^{\top}\theta^* + \epsilon_{ij},$$

where $\boldsymbol{\theta}^* = \boldsymbol{\theta} + \Sigma_{\mathbf{Z}}^{-1}\Sigma_{\mathbf{Z},\mathbf{X}}\beta$ and $\mu^* = \mu + \boldsymbol{\mu}_{\mathbf{Z}}^{\top}\boldsymbol{\Sigma}_{\mathbf{Z}}^{-1}\boldsymbol{\Sigma}_{\mathbf{Z},X}\beta$. Now,

$$X_{ij}^*|\alpha_i, \gamma_i \sim N(\gamma_i, \sigma_X^{*2}),$$

where $\sigma_X^{*2} = \sigma_X^2 - \boldsymbol{\Sigma}_{X,\mathbf{Z}}\boldsymbol{\Sigma}_{\mathbf{Z}}^{-1}\boldsymbol{\Sigma}_{\mathbf{Z},\mathbf{X}} = \sigma_X^2(1 - R^2)$ and R^2 is the coefficient of determination for the regression of X_{ij} on $\mathbf{Z}_{ij}$. It then follows from part a) that the bias in the estimate of β is

$$\frac{\rho\sigma_\alpha\sigma_\gamma}{\sigma_X^{*2}} \left\{ 1 + \frac{\sigma_\gamma^2}{\sigma_X^{*2}} + \frac{(n-1)\sigma_\alpha^2}{\sigma_\epsilon^2} \right\}^{-1}.$$

E Simulations on the Impact of Estimating η

We assess the impact of estimating the nuisance parameter η on the bias of the RE estimator. Specifically, we compare the empirical bias of $\hat{\beta}_{\text{RE}}$, computed from simulated data, with the theoretical bias predicted by the asymptotic formula in Proposition 1. The data were generated following the procedure described in Section 4 with $m = 100$ clusters and $n = 100$ observations per cluster, and default parameters $\sigma_\epsilon^2 = 1$, $\sigma_\alpha^2 = 1$, $\sigma_\gamma^2 = 0.5$, and

$\sigma_X^2 = 0.5$. We then varied σ_ϵ^2, σ_α^2, and σ_γ^2 individually while holding the remaining parameters fixed. The empirical results were based on 10,000 replicated data sets. As shown in Figures S1–S3, when σ_α^2 is relatively large compared with σ_ϵ^2 and σ_γ^2, the empirical bias and the theoretical bias are approximately equal as ρ changes. However, when σ_α^2 is relatively small (e.g., 0.5) compared with σ_ϵ^2 and σ_γ^2, as we increase σ_γ^2 from 0.5 to 4 or increase σ_ϵ^2 from 1 to 8: the discrepancy between the empirical and theoretical bias grows. We argue that this is caused by the underestimation of σ_α when σ_γ or σ_ϵ is large, leading to a larger empirical $\lambda(\eta)$ compared to its theoretical value.

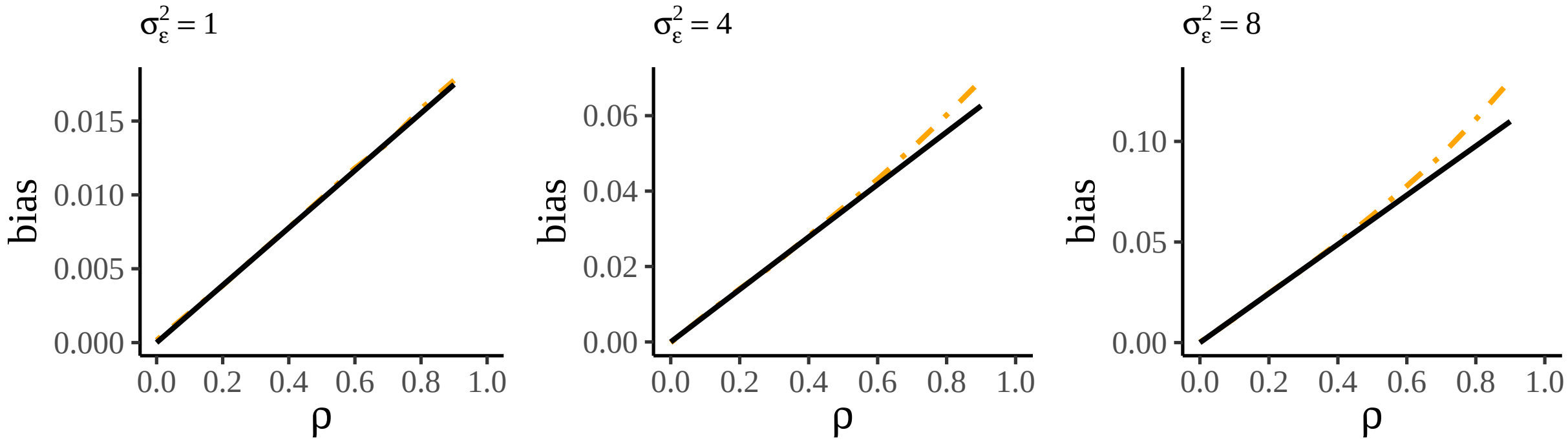

(a) $\sigma_\alpha^2 = 0.5$

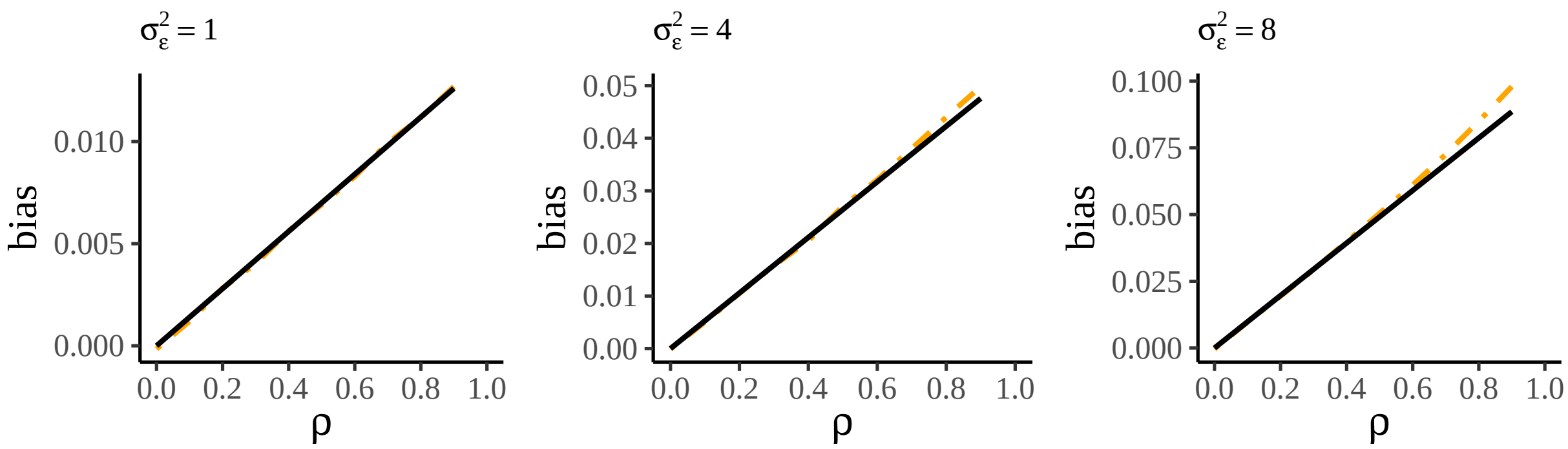

(b) $\sigma_\alpha^2 = 1$

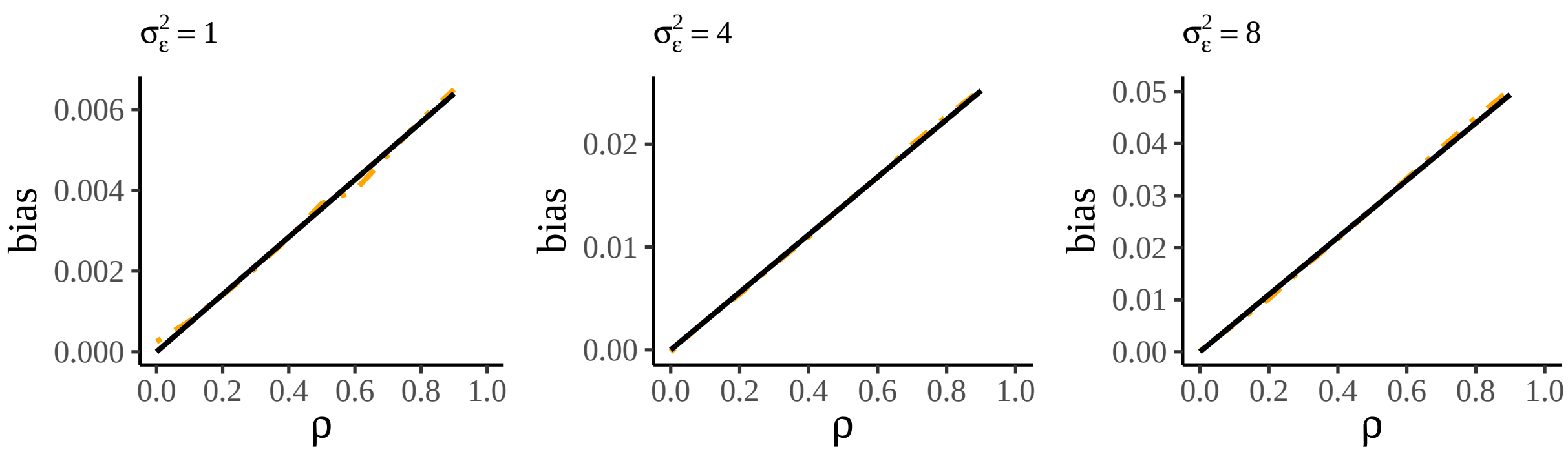

(c) $\sigma_\alpha^2 = 4$

Figure S1. Comparison between the empirical bias (orange), with estimated η, and theoretical bias (black), assuming η is known, of random effects models in estimating β, as σ_α^2 varies from 0.5 to 4 (top to bottom), and σ_ϵ^2 varies from 1 to 8 (left to right). The other parameters are set as follows: number of clusters $m = 100$, cluster size $n = 100$, $\sigma_X^2 = 0.5$, and $\sigma_\gamma^2 = 0.5$.

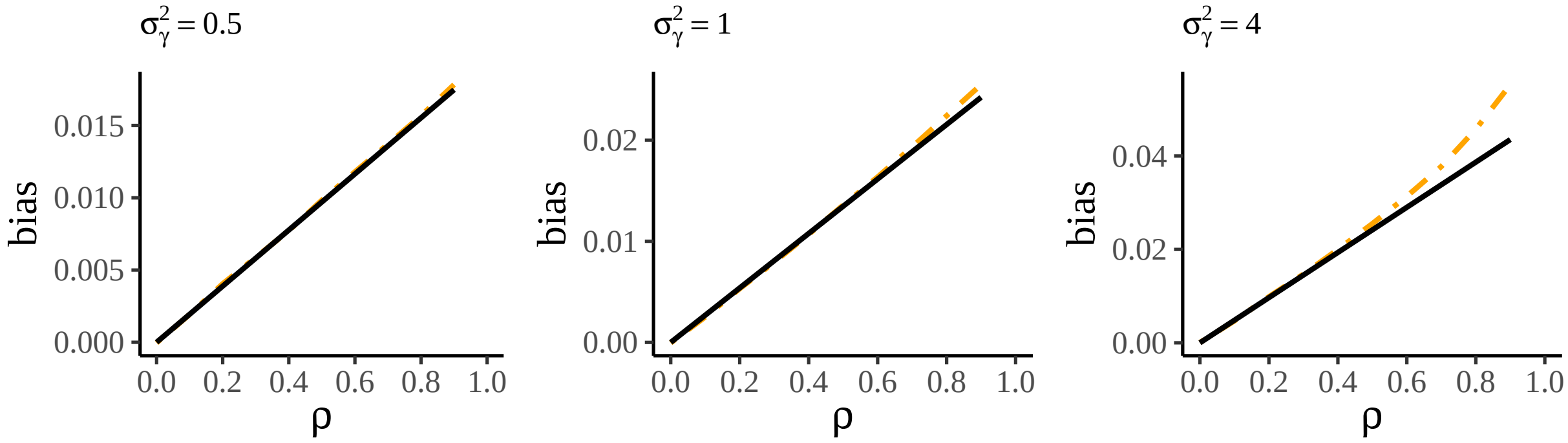

(a) $\sigma_\alpha^2 = 0.5$

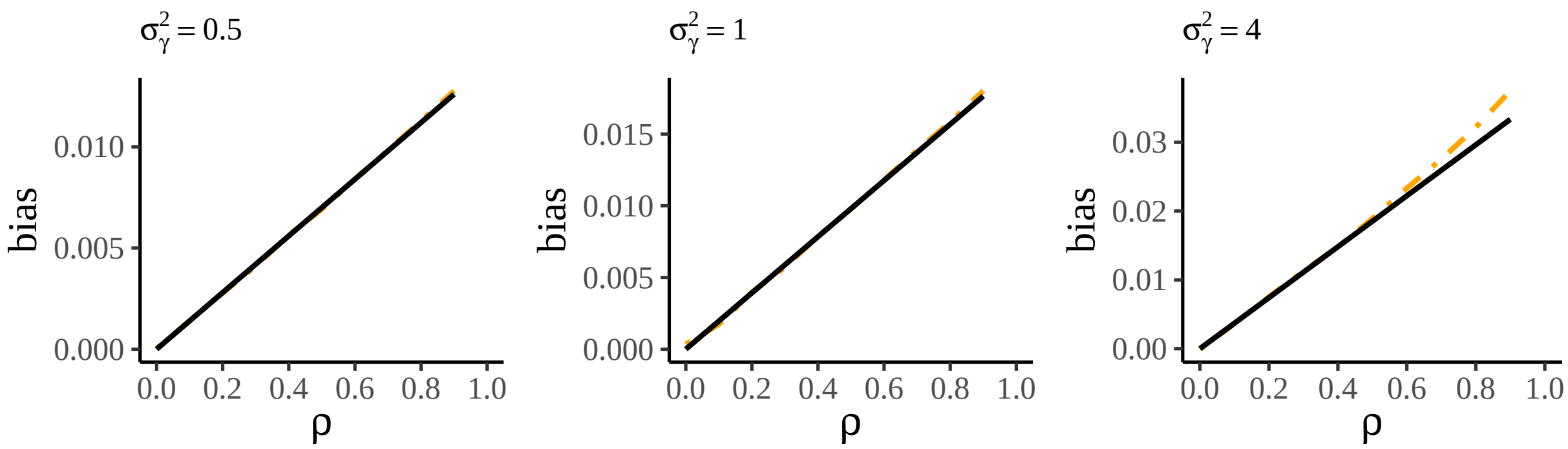

(b) $\sigma_\alpha^2 = 1$

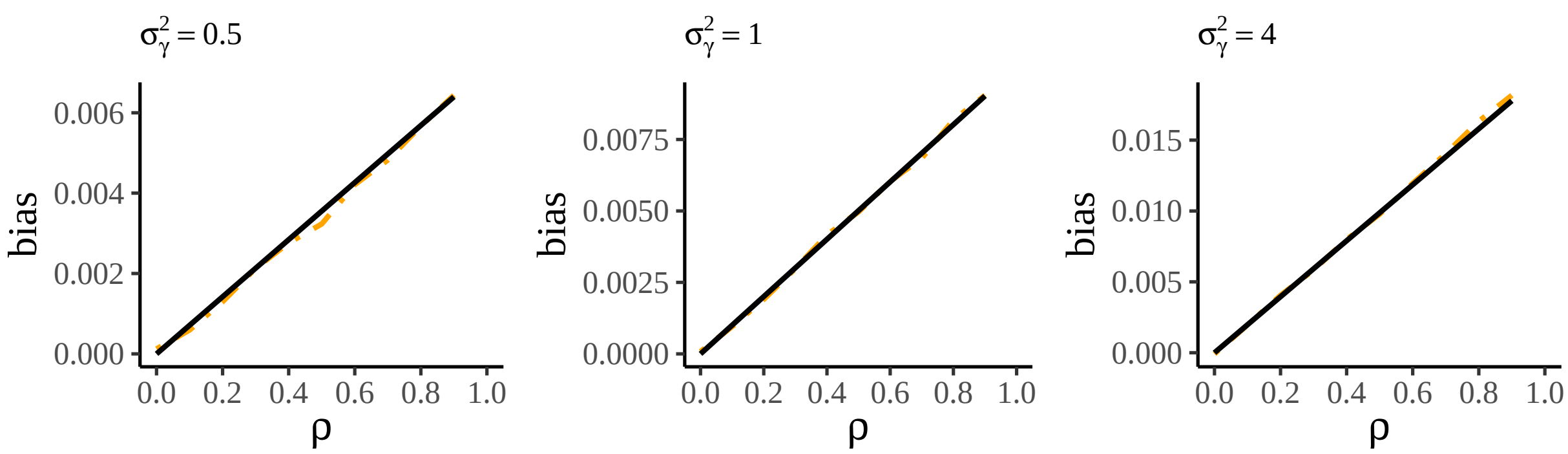

(c) $\sigma_\alpha^2 = 4$

Figure S2. Comparison between the empirical bias (orange), with estimated η, and theoretical bias (black), assuming η is known, of random effects models in estimating β, as σ_α^2 varies from 0.5 to 4 (top to bottom), and σ_γ^2 varies from 0.5 to 4 (left to right). The other parameters are set as follows: number of clusters $m = 100$, cluster size $n = 100$, $\sigma_X^2 = 0.5$, and $\sigma_\epsilon^2 = 1$.

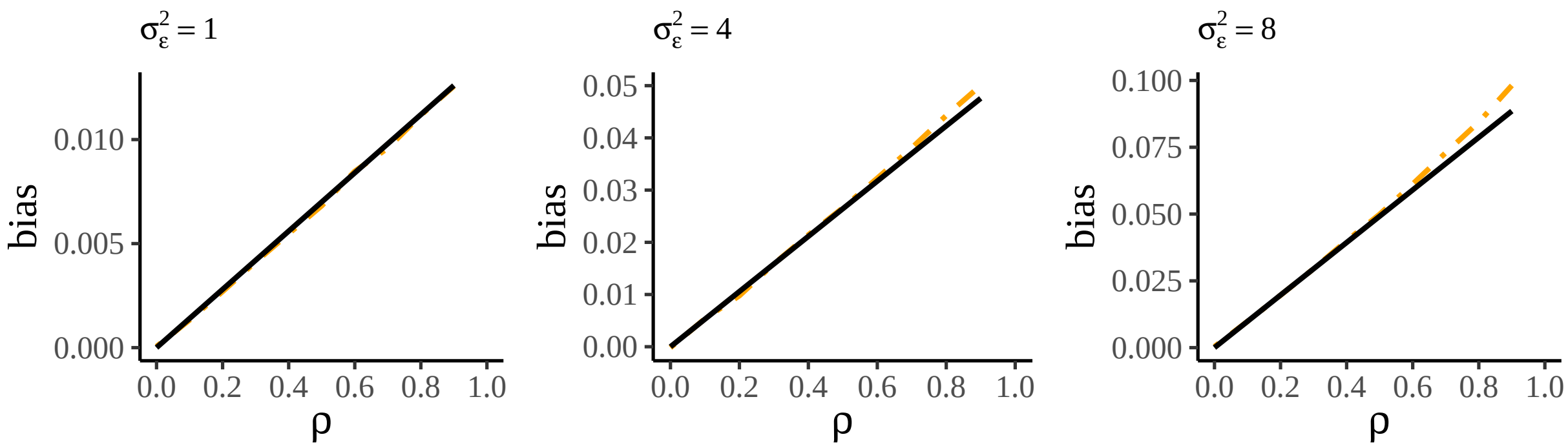

(a) $\sigma_\gamma^2 = 0.5$

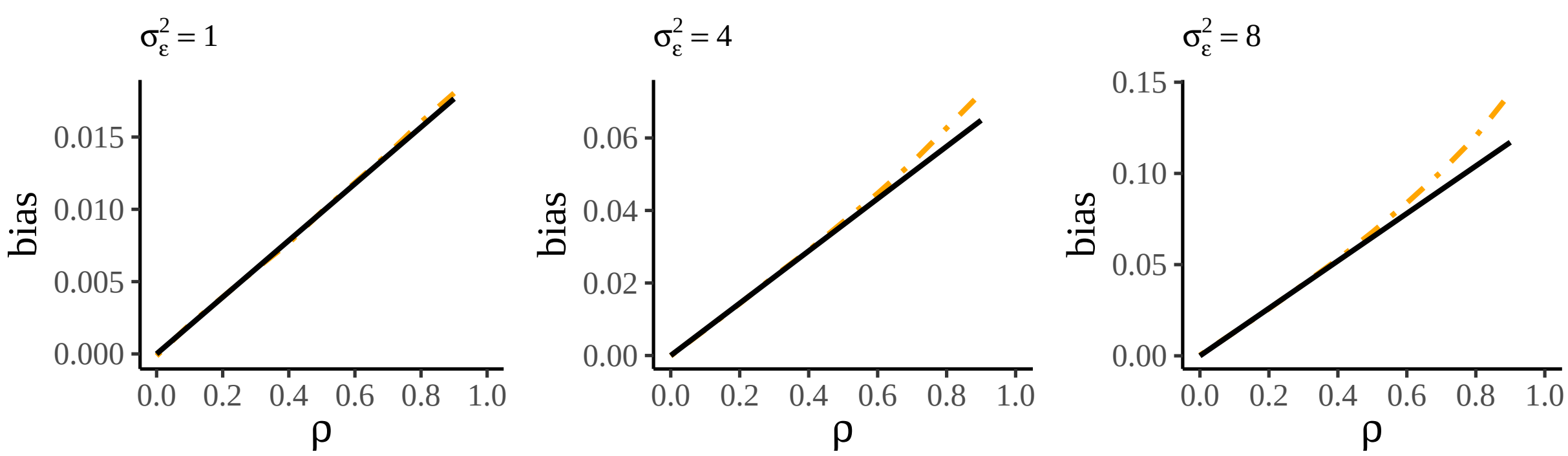

(b) $\sigma_\gamma^2 = 1$

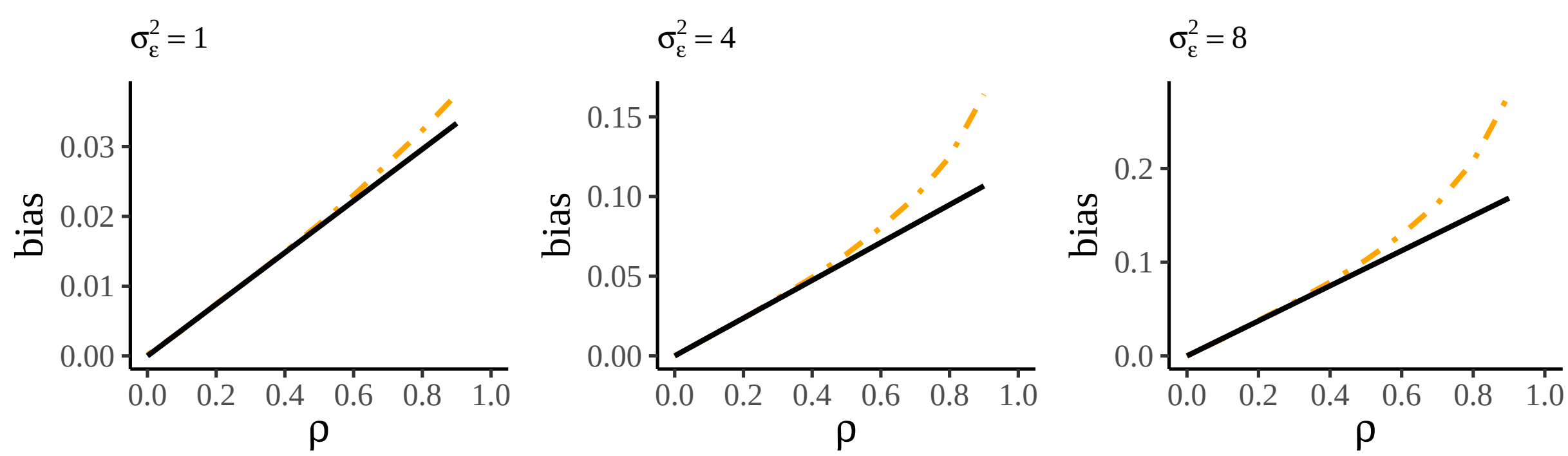

(c) $\sigma_\gamma^2 = 4$

Figure S3. Comparison between the empirical bias (orange), with estimated η, and theoretical bias (black), assuming η is known, of random effects models in estimating β, as σ_γ^2 varies from 0.5 to 4 (top to bottom), and σ_ϵ^2 varies from 1 to 8 (left to right). The other parameters are set as follows: number of clusters $m = 100$, cluster size $n = 100$, $\sigma_X^2 = 0.5$, and $\sigma_\alpha^2 = 1$.

F Additional Simulation Studies for Evaluation of β Estimators

In this appendix, we present results from additional simulation experiments designed to assess the robustness of the main findings to departures from the baseline simulation design

described in Section 4. Unless otherwise noted, all settings not explicitly varied follow the baseline configuration with $m = 40$, $n = 50$, $\beta = 1$, $\sigma_\alpha^2 = 1$, $\sigma_\gamma^2 = 0.5$, $\sigma_X^2 = 0.5$, and $\sigma_\epsilon^2 = 16$.

F.1 Heavy-tailed outcome distributions

To assess robustness to violations of the normality assumption, we replaced the Gaussian error ϵ_{ij} with a scaled Student's t distribution. The scaling was chosen so that the error variance remained σ_ϵ^2, ensuring that the signal-to-noise ratio is directly comparable to the baseline Gaussian setting. We examined three degrees of freedom, $\nu = 3, 5$, and 30, representing strongly heavy-tailed, moderately heavy-tailed, and approximately normal error distributions, respectively. Results were summarized using empirical bias and RMSE based on 5,000 replicated data sets under each configuration.

Figure S4 shows that the qualitative conclusions remain unchanged under heavy-tailed outcome distributions. The FE estimator of β remains approximately unbiased across all values of ρ and all degrees of freedom considered, whereas the RE estimator continues to exhibit bias that increases with $|\rho|$, consistent with the theoretical bias expression. Varying the tail behavior of the error distribution has little impact on these qualitative patterns, indicating that the comparative behavior of the FE and RE estimators is robust to moderate departures from normality.

F.2 Binary predictor

To assess robustness to the distributional form of the covariate, we replaced the continuous predictor with a binary covariate. We generated a latent continuous covariate with cluster-specific mean and then dichotomized it using a threshold chosen to achieve a prespecified marginal prevalence. Specifically, within each cluster we simulated a latent variable and set $X_{ij} = 1$ if it exceeded a cutoff and $X_{ij} = 0$ otherwise; the cutoff was calibrated so that $\Pr(X_{ij} = 1) = \pi_x$ marginally. We considered $\pi_x \in \{0.3, 0.5, 0.7\}$ to represent low, moderate, and high prevalence of the binary predictor.

Figure S5 shows that replacing the continuous predictor with a binary covariate does not materially alter the comparative behavior of the FE and RE estimators. The FE estimator of β remains approximately unbiased across all values of ρ, whereas the RE estimator exhibits bias that increases with $|\rho|$. Varying the marginal prevalence from $\pi_x = 0.3$ to $\pi_x = 0.7$ has little effect on these qualitative patterns, suggesting that the conclusions are robust to the distributional form of the predictor.

F.3 More complex random effect structures

To examine the sensitivity of β estimation to the random effect structure, we treated cluster 1 as the cluster of interest and fixed α_1 at prespecified values (0, 1.5, and 3), while all remaining clusters were generated according to the baseline procedure. To maintain consistency with the joint cluster-level structure, we generated the cluster-1 covariate mean from its conditional distribution given α_1,

$$\gamma_1 \mid \alpha_1 \sim \mathcal{N}\left(\rho \frac{\sigma_\gamma}{\sigma_\alpha} \alpha_1,\ (1 - \rho^2)\sigma_\gamma^2\right). \tag{1}$$

Note that this simulation design parallels the one used in Section 4.2 for evaluating α estimators; here we focus on the estimation of β under the same data-generating mechanism.

Figure S6 shows that fixing the first cluster effect at different magnitudes does not materially affect the estimation of β. The FE estimator remains approximately unbiased across all values of ρ, whereas the RE estimator continues to exhibit bias that increases with $|\rho|$. Varying the magnitude of α_1 from 0 to 3 has little impact on these qualitative patterns, indicating that the estimation of β is largely insensitive to the magnitude of individual cluster effects.

F.4 Unbalanced cluster sizes

To investigate the impact of unequal cluster sizes, we generated the cluster size n_i from a log-normal distribution rather than fixing it. The parameters of the log-normal distribution were chosen so that the expected cluster size equaled a prespecified target value $n_{\text{target}} = 50$. Specifically, if $\log(n_i) \sim \mathcal{N}(\mu_n, {\sigma_n}^2)$, we set

$$\mu_n = \log(n_{\text{target}}) - \frac{{\sigma_n}^2}{2}, \tag{2}$$

which ensures $\mathbb{E}(n_i) = n_{\text{target}}$. The realized n_i values were rounded to the nearest integer and truncated below at one.

We considered three levels of between-cluster imbalance by varying σ_n as 0.1, 0.6, and 1.0, corresponding to coefficients of variation $\text{CV}(n_i) = \sqrt{e^{\sigma_n^2} - 1}$ of approximately 0.10, 0.66, and 1.31. In particular, when $\sigma_n = 1.0$, the realized cluster sizes can be extremely heterogeneous, with the smallest clusters containing as few as one observation while the largest may exceed 5,000 observations.

Figure S7 shows that allowing cluster sizes to vary does not materially change the comparative behavior of the FE and RE estimators. The qualitative patterns remain consistent with the baseline simulations across all levels of imbalance, indicating that the conclusions are robust to substantial heterogeneity in cluster sizes.

F.5 Multiple endogenous covariates

To extend the univariate setting to the multivariate case, we considered $p = 2$ covariates while preserving the same hierarchical structure. Let $\boldsymbol{\gamma}_i = (\gamma_{i1}, \dots, \gamma_{ip})^\top$ denote the vector of underlying within-cluster means. We generated $(\alpha_i, \boldsymbol{\gamma}_i)$ jointly from a multivariate normal distribution,

$$\begin{pmatrix} \alpha_i \\ \boldsymbol{\gamma}_i \end{pmatrix} \overset{i.i.d.}{\sim} \mathcal{N}\left(\begin{pmatrix} 0 \\ \mathbf{0} \end{pmatrix}, \begin{pmatrix} \sigma_\alpha^2 & \rho\sigma_\alpha\sigma_\gamma \, \mathbf{1}_p^\top \\ \rho\sigma_\alpha\sigma_\gamma \, \mathbf{1}_p & \boldsymbol{\Sigma}_\gamma \end{pmatrix} \right), \tag{3}$$

where each element of $\text{Cov}(\boldsymbol{\gamma}_i, \alpha_i)$ equals $\rho\sigma_\alpha\sigma_\gamma$, and

$$\boldsymbol{\Sigma}_\gamma = \sigma_\gamma^2 \big((1 - \rho_\gamma)\mathbf{I}_p + \rho_\gamma \mathbf{1}_p \mathbf{1}_p^\top \big), \tag{4}$$

so that $\text{Var}(\gamma_{ik}) = \sigma_\gamma^2$ and $\text{Corr}(\gamma_{ik}, \gamma_{i\ell}) = \rho_\gamma$ for $k \neq \ell$. Conditional on $(\boldsymbol{\gamma}_i, \alpha_i)$, we generated n observations within cluster i as

$$\mathbf{X}_{ij} \mid \boldsymbol{\gamma}_i, \alpha_i \overset{i.i.d.}{\sim} \mathcal{N}(\boldsymbol{\gamma}_i, \sigma_X^2 \mathbf{I}_p), \tag{5}$$

$$\epsilon_{ij} \overset{i.i.d.}{\sim} \mathcal{N}(0, \sigma_\epsilon^2), \tag{6}$$

and $Y_{ij} = \alpha_i + \mathbf{X}_{ij}^\top \boldsymbol{\beta} + \epsilon_{ij}$.

Because α_i is assumed to have equal correlation ρ with every component of $\boldsymbol{\gamma}_i$, the joint covariance matrix is positive definite only when $\rho^2 < (1 + (p-1)\rho_\gamma)/p$. For $p = 2$ this simplifies to $\rho^2 < (1+\rho_\gamma)/2$. We examined three settings: $\rho_\gamma \in \{0, 0.2, 0.4\}$, which permit $|\rho|$ up to approximately 0.71, 0.77, and 0.84, respectively. In each setting we varied ρ from -0.6 to 0.6. Simulation performance was summarized by averaging bias and RMSE across the p regression coefficients.

Figure S8 shows that the key findings from the univariate case extend to the setting with $p = 2$ endogenous covariates. The FE estimator of $\boldsymbol{\beta}$ remains approximately unbiased across all values of ρ and all levels of inter-covariate correlation, whereas the RE estimator exhibits bias that increases with $|\rho|$. Increasing ρ_γ from 0 to 0.4 has little impact on the bias or RMSE of either estimator, suggesting that the degree of dependence among the covariates does not materially alter the bias–variance trade-off between FE and RE estimation.

F.6 Settings Where FE and RE Lead to Different Scientific Conclusions

The preceding simulations demonstrate that the RE estimator of β carries a constant positive bias when $\rho > 0$. A natural question is whether this bias is large enough to alter the scientific conclusion drawn from the data. To address this, we designed a simulation in which the true effect β varies continuously from negative to positive while the correlation ρ is held fixed, and we evaluated each estimator using three standard frequentist criteria: confidence interval coverage, mean squared error, and Type I error calibration.

The data-generating mechanism follows the baseline design in Section 4 in the main manuscript, with parameter choices intended to produce a setting where the RE bias is large relative to the magnitude of β. We set $m = 40$ clusters, $n_i = 20$ observations per cluster, $\sigma_\alpha = 2$, $\sigma_\gamma = \sqrt{0.5}$, $\sigma_X = \sqrt{0.5}$, $\sigma_\epsilon = 1$, and $\rho = 0.6$. Under these settings, the theoretical RE bias from Proposition 1 is approximately $+0.022$, regardless of the true value of β.

We varied β over the grid $\{-0.15, -0.125, \ldots, 0.125, 0.15\}$. For each value of β, we generated 5,000 replicated datasets, fit both the FE and RE models, and recorded the point estimate $\hat{\beta}$, its standard error, and the corresponding 95% Wald confidence interval. We then computed three quantities for each method: the empirical coverage probability of the 95% confidence interval (the proportion of replications in which the interval contains the true β), the mean squared error decomposed into bias^2 and variance, and the rejection rate for testing H_0: $\beta = 0$ at the 5% significance level.

Figure S9 presents the results in three panels.

Coverage (Panel A). The FE estimator maintains coverage at approximately 95% across the entire range of β, confirming that its confidence intervals are correctly calibrated. The RE estimator, by contrast, achieves only 91–93% coverage uniformly, falling 2–4 percentage points below the nominal level. This coverage deficit arises because the constant positive bias shifts the center of the RE confidence interval away from the true β, causing the interval to miss the true value more often than the nominal rate allows. Notably, the RE undercoverage is not confined to a narrow region of the parameter space;

it persists across all values of β, indicating a systematic miscalibration of the RE inference. An applied researcher relying on RE confidence intervals would therefore understate the uncertainty of the estimated exposure-outcome association for every covariate in the model, regardless of its true effect size.

Mean Squared Error (Panel B). The MSE of the FE estimator consists entirely of variance (light shading), as its bias is negligible. The RE estimator has slightly lower variance, reflecting the efficiency gain from partial pooling. However, it also carries a constant bias^2 component (dark shading) that more than offsets the variance reduction. As a result, the total MSE of the RE estimator exceeds that of the FE estimator across the entire range of β. This result demonstrates that the well-known efficiency advantage of RE estimation is negated when the RE assumption is violated: the bias-variance tradeoff, which is often cited as the rationale for preferring RE, turns unfavorable once the correlation between cluster effects and covariates is non-negligible.

Rejection Rate and Type I Error (Panel C). At $\beta = 0$, the FE estimator rejects H_0: $\beta = 0$ at approximately the nominal 5% level, whereas the RE estimator rejects at a substantially higher rate, reflecting an inflated Type I error driven by the positive bias. As $|\beta|$ increases, both methods gain power, but the RE rejection curve is shifted to the right relative to FE: the RE model has lower power for negative β (where the positive bias attenuates the estimate toward zero) and apparently higher power for positive β (where the bias inflates the estimate away from zero). This apparent power advantage of RE on the positive side is not genuine sensitivity; it is the same bias that causes the inflated Type I error at $\beta = 0$ and the coverage deficit in Panel (A), manifesting as excess rejections whenever the bias direction coincides with the sign of the true effect.

Taken together, these results demonstrate that the FE estimator dominates the RE estimator on all three standard evaluation criteria when the RE assumption is violated. The FE estimator provides correctly calibrated confidence intervals, lower mean squared error, and proper Type I error control. The RE estimator, while offering a modest variance reduction, pays for it with a bias that distorts coverage, inflates MSE, and produces systematically misleading rejection rates. For the consistent estimation of $\boldsymbol{\beta}$ in settings where cluster effects may be correlated with covariates, the FE approach is the only framework that provides reliable frequentist inference.

G Additional Results: Provider Profiling

This appendix presents the full provider-level flagging results for the kidney transplant center profiling application described in Section 5.2 of the main paper. While the main text focuses on the estimation of the regression coefficients $\boldsymbol{\beta}$, the choice of modeling framework also affects the predicted center effects and, consequently, the regulatory classifications assigned to individual providers. We present these results not to advocate for a particular flagging methodology but to document the magnitude of the disagreement across standard approaches and to highlight the judgment that is required when translating model output into policy decisions.

Table S1 cross-tabulates the regulatory outlier classifications ("Worse," "Average," or "Better") across the five modeling frameworks. The extent of the disagreement is striking. Panel A shows that switching from FE to RE alters the classification of 13 of the 179 centers: the RE model downgrades 4 centers from "Better" to "Average" and reclassifies 9 "Average" centers as "Worse." Panels B through D show that introducing the CRE specification produces progressively larger reclassifications. Under the fully specified CRE III (Panel D), 68 of the 179 centers (38.0%) receive a different regulatory designation than under FE, including 5 centers that move from "Worse" to "Better" and 1 that moves from "Better" to "Worse."

The disagreement is not limited to comparisons against the FE baseline. Panels E through H compare the RE and CRE models against each other. Thirty centers are reclassified between CRE I and CRE II (Panel G), and 59 between CRE II and CRE III (Panel H). These results demonstrate that the flagging outcomes are sensitive not only to the broad choice between FE and RE but also to the specific set of contextual means included in the CRE specification.

Determining which set of flagging outcomes is "correct" is not straightforward, as the answer depends on considerations that go beyond coefficient estimation: the choice of null hypothesis (whether a center is compared to the population mean, the median, or an external standard), the desired balance between sensitivity and specificity in outlier detection, the role of shrinkage in stabilizing estimates for small centers, and the policy objectives of the profiling program. These are inherently judgment calls that require input from both statisticians and clinical stakeholders. The present paper establishes that the bias in $\boldsymbol{\beta}$ is a first-order contributor to the disagreement documented above, but a complete framework for provider testing and flagging under endogenous confounding is the subject of separate ongoing work.

Table S1. Cross-tabulation of transplant center regulatory flagging status across statistical models ($n = 179$ centers with ≥ 20 patients; OPTN, July 2019–June 2021; $N = 27{,}891$ patients). Each 3×3 sub-panel compares the outlier classification assigned by the row method against the column method, based on discharge eGFR risk-adjusted for 68 patient-level covariates. Bold diagonal entries denote concordant classifications. W = Worse, A = Average, B = Better. Models: FE = Fixed Effects, RE = Random Effects, CRE I = additionally adjusting for center-mean donor eGFR, CRE II = additionally adjusting for 15 additional center-level covariate means (16 in total, including donor eGFR), CRE III = additionally adjusting for all 68 center-level covariate means.

Panel	Row	W	A	B	Panel	Row	W	A	B
A: FE–RE	Worse	**57**	0	0	*E: RE–CRE I*	Worse	**51**	14	1
	Average	9	**66**	0		Average	11	**57**	2
	Better	0	4	**43**		Better	0	8	**35**
B: FE–CRE I	Worse	**45**	12	0	*F: RE–CRE III*	Worse	**35**	25	6
	Average	17	**55**	3		Average	8	**53**	9
	Better	0	12	**35**		Better	0	17	**26**
C: FE–CRE II	Worse	**37**	20	0	*G: CRE I–II*	Worse	**48**	14	0
	Average	16	**56**	3		Average	5	**69**	5
	Better	0	13	**34**		Better	0	6	**32**
D: FE–CRE III	Worse	**30**	22	5	*H: CRE II–III*	Worse	**33**	19	1
	Average	12	**54**	9		Average	10	**63**	16
	Better	1	19	**27**		Better	0	13	**24**

*REFERENCES

[1] Mundlak Y. On the pooling of time series and cross section data. *Econometrica.* 1978;46:69–85.

[2] Neuhaus JM, Kalbfleisch JD. Between-and within-cluster covariate effects in the analysis of clustered data. *Biometrics.* 1998:638–645.

[3] Scott AJ, Holt D. The effect of two-stage sampling on ordinary least squares methods. *Journal of the American Statistical Association.* 1982;77(380):848–854.

[4] Shen L, Shao J, Park S, Palta M. Between-and within-cluster covariate effects and model misspecification in the analysis of clustered data. *Statistica Sinica.* 2008;18(2):731–748.

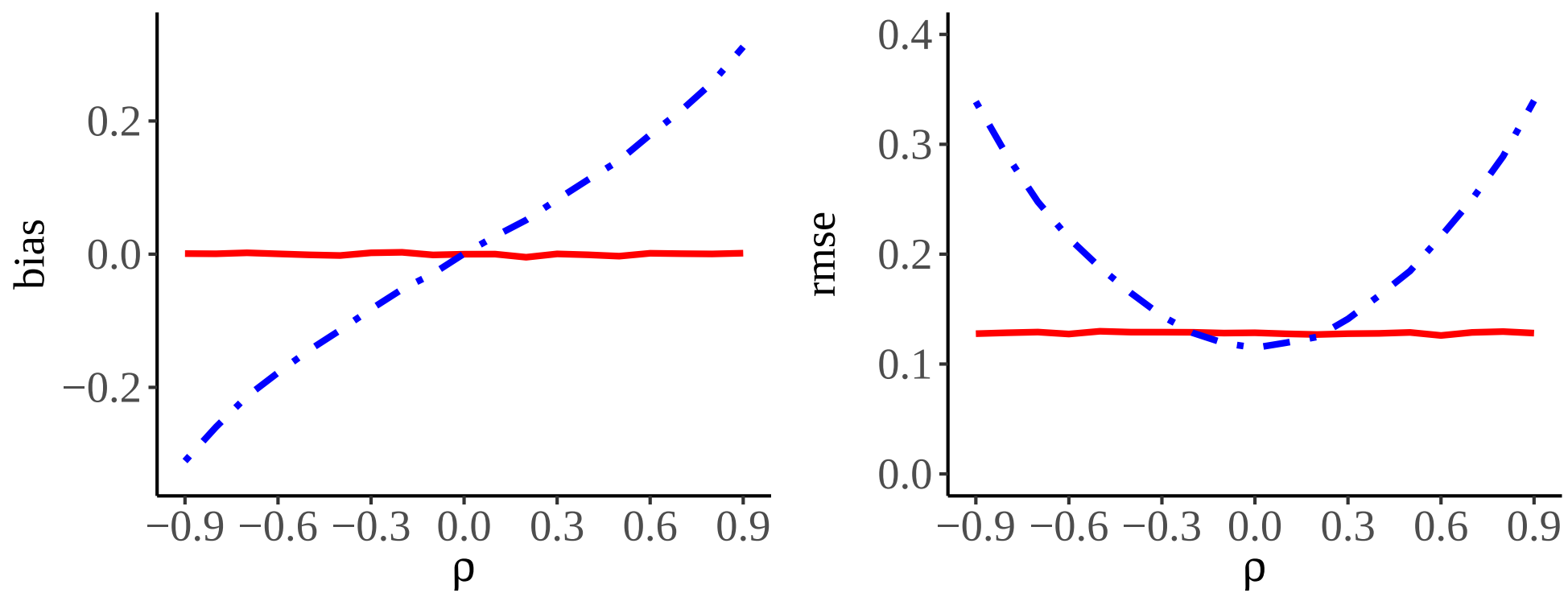

(a) $\nu = 30$

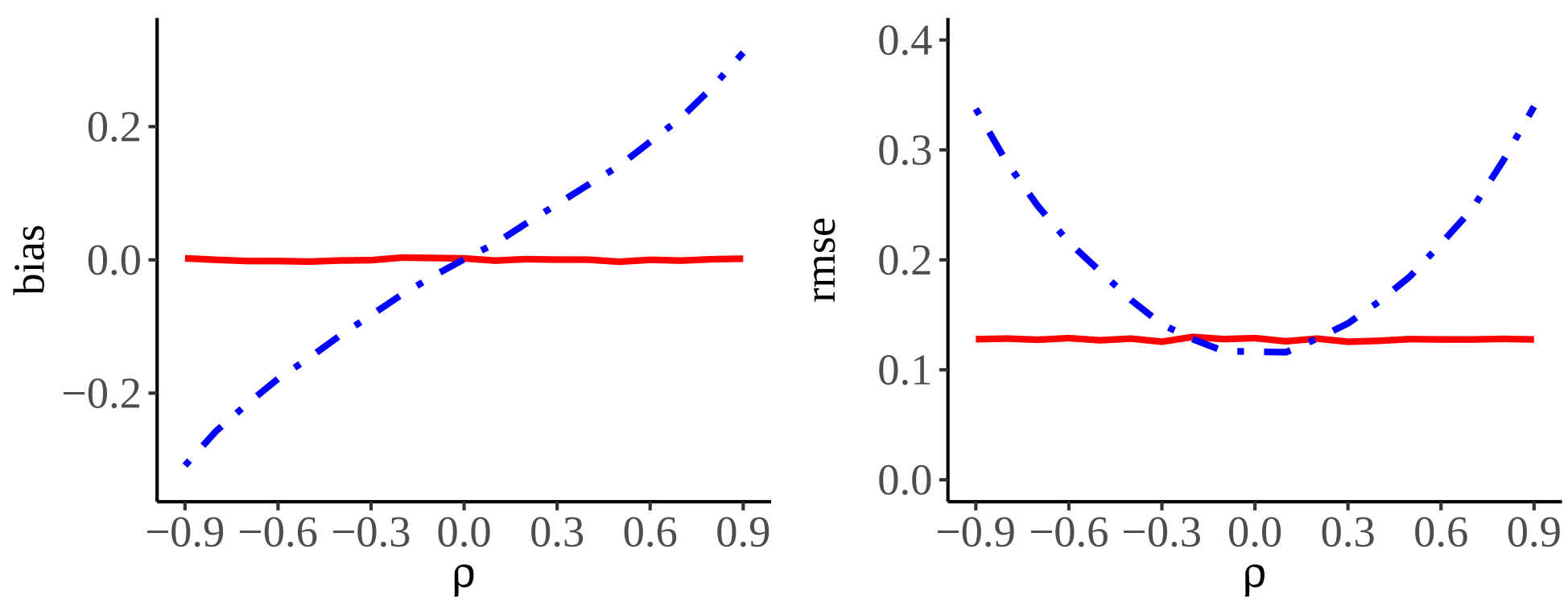

(b) $\nu = 5$

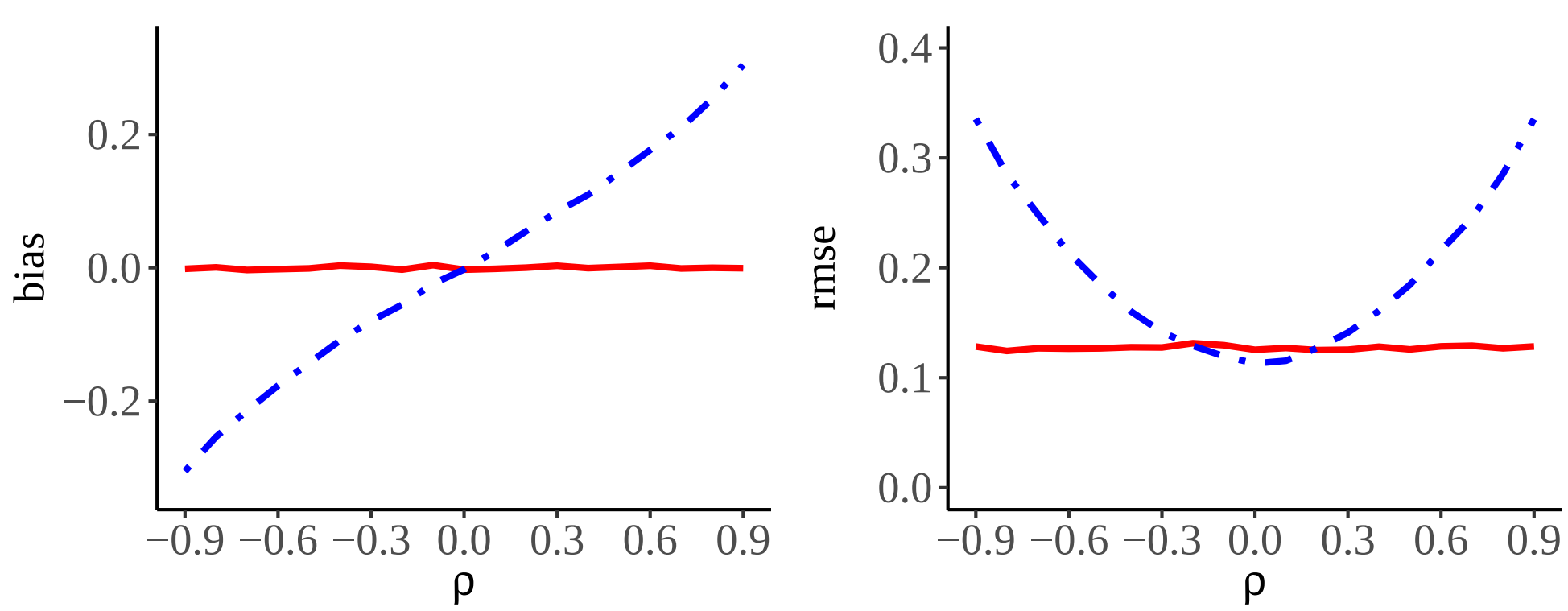

(c) $\nu = 3$

Figure S4. Empirical bias and RMSE for estimating β under error distributions with different tail behaviors. Panels correspond to t_ν errors with $\nu = 30$, 5, and 3, representing approximately normal, moderately heavy-tailed, and strongly heavy-tailed error distributions, respectively. Results are shown across values of ρ, comparing the fixed-effects model (red solid curves) and the random-effects model (blue dashed curves).

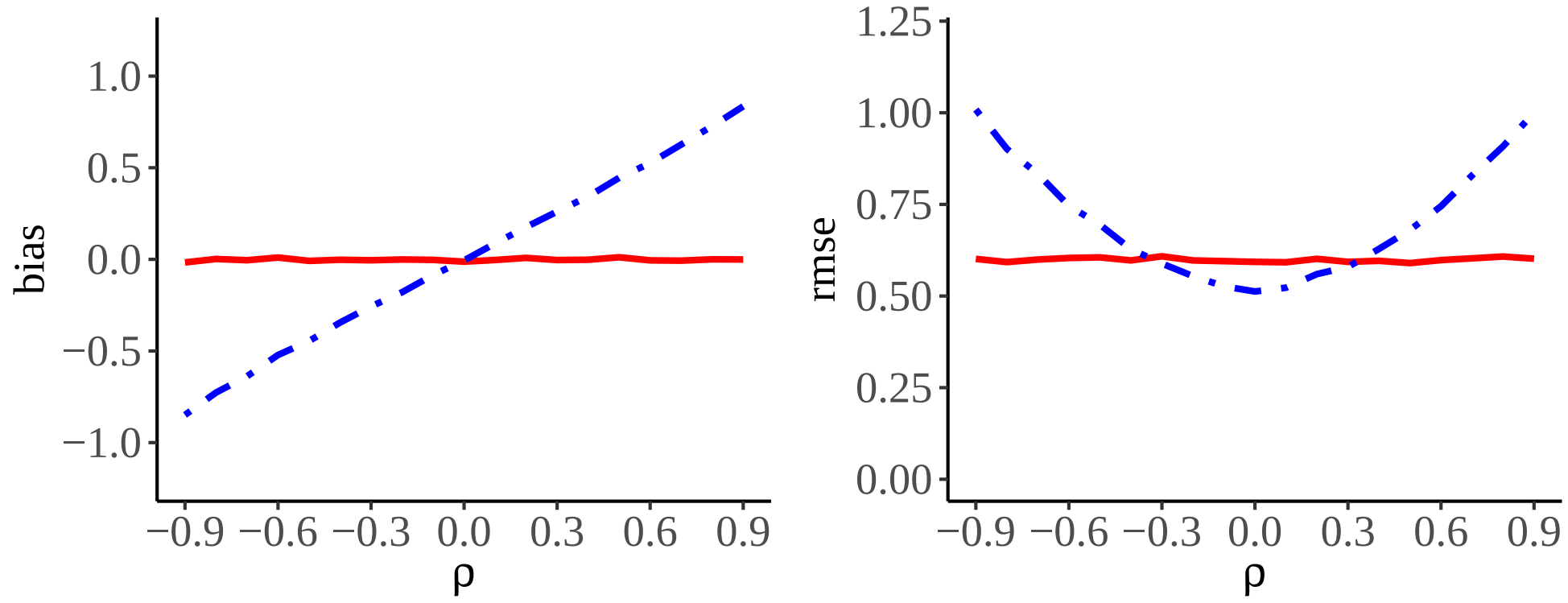

(a) $\pi_x = 0.3$

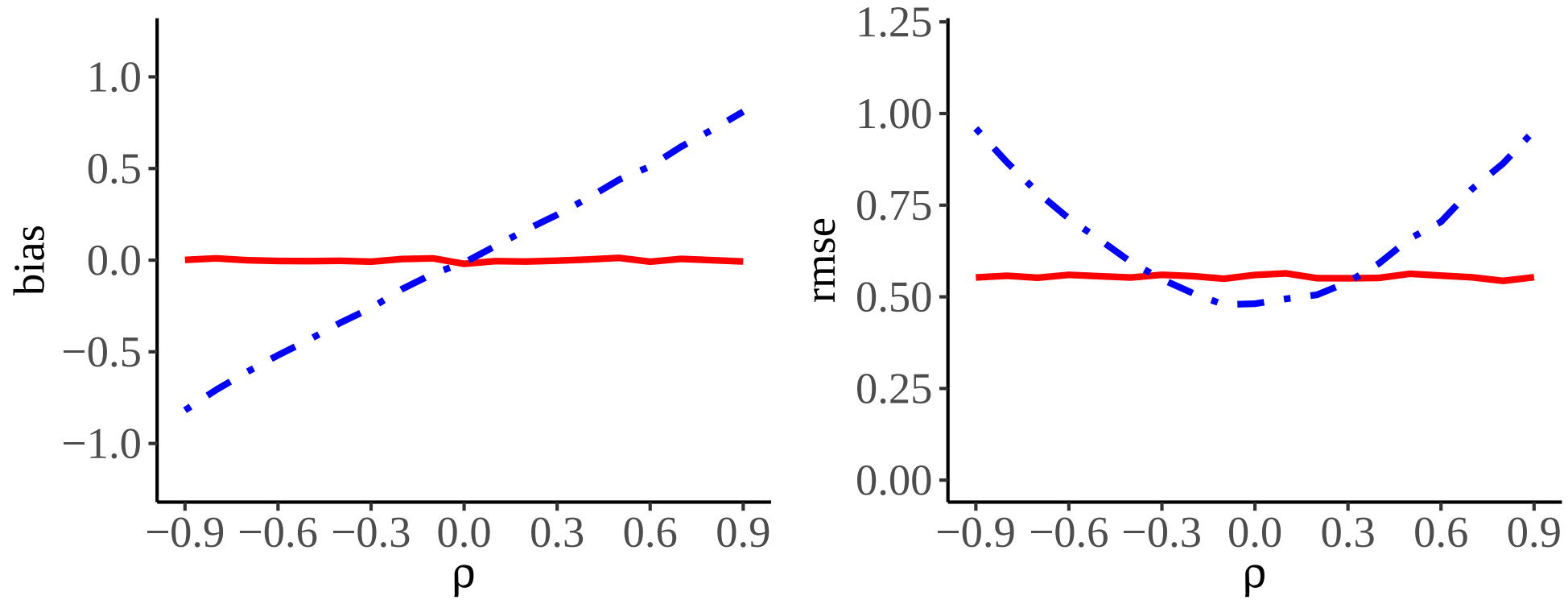

(b) $\pi_x = 0.5$

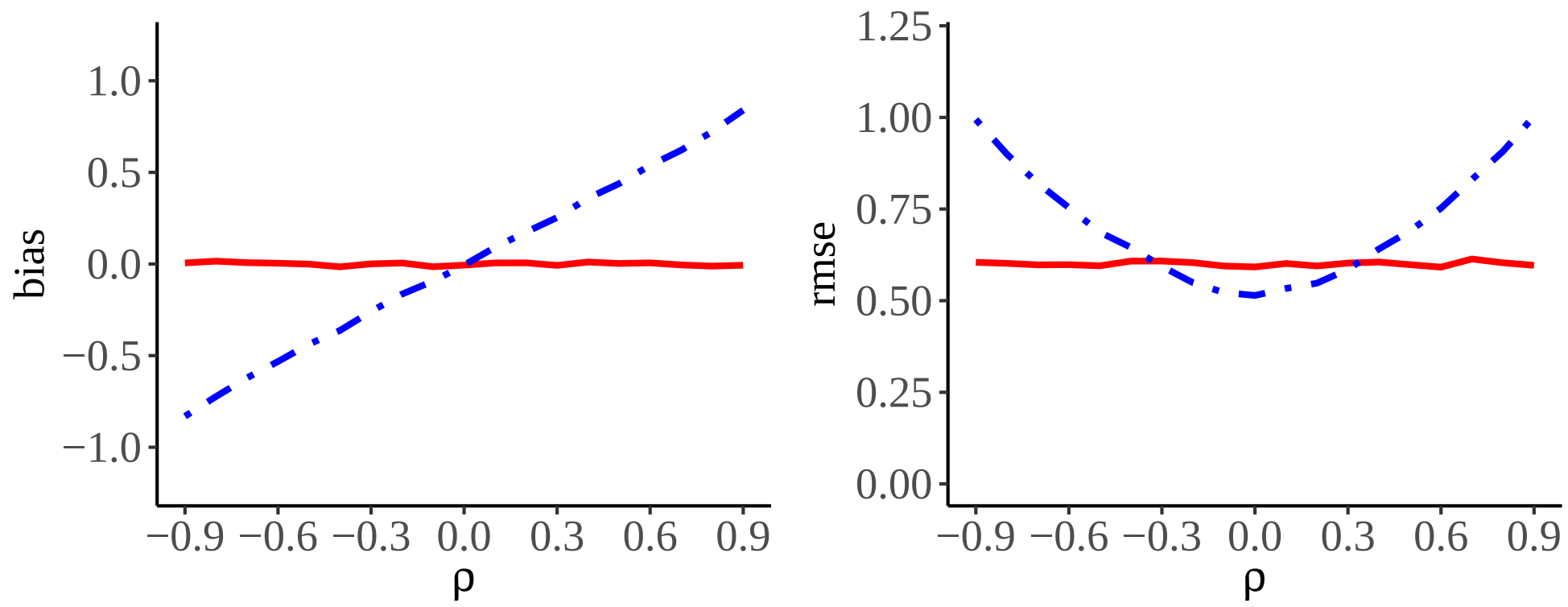

(c) $\pi_x = 0.7$

Figure S5. Empirical bias and RMSE for estimating β across values of ρ, comparing the fixed-effects model (red solid curves) and the random-effects model (blue dashed curves). Panels correspond to binary predictors with marginal prevalence $\pi_x = 0.3$, 0.5, and 0.7, respectively.

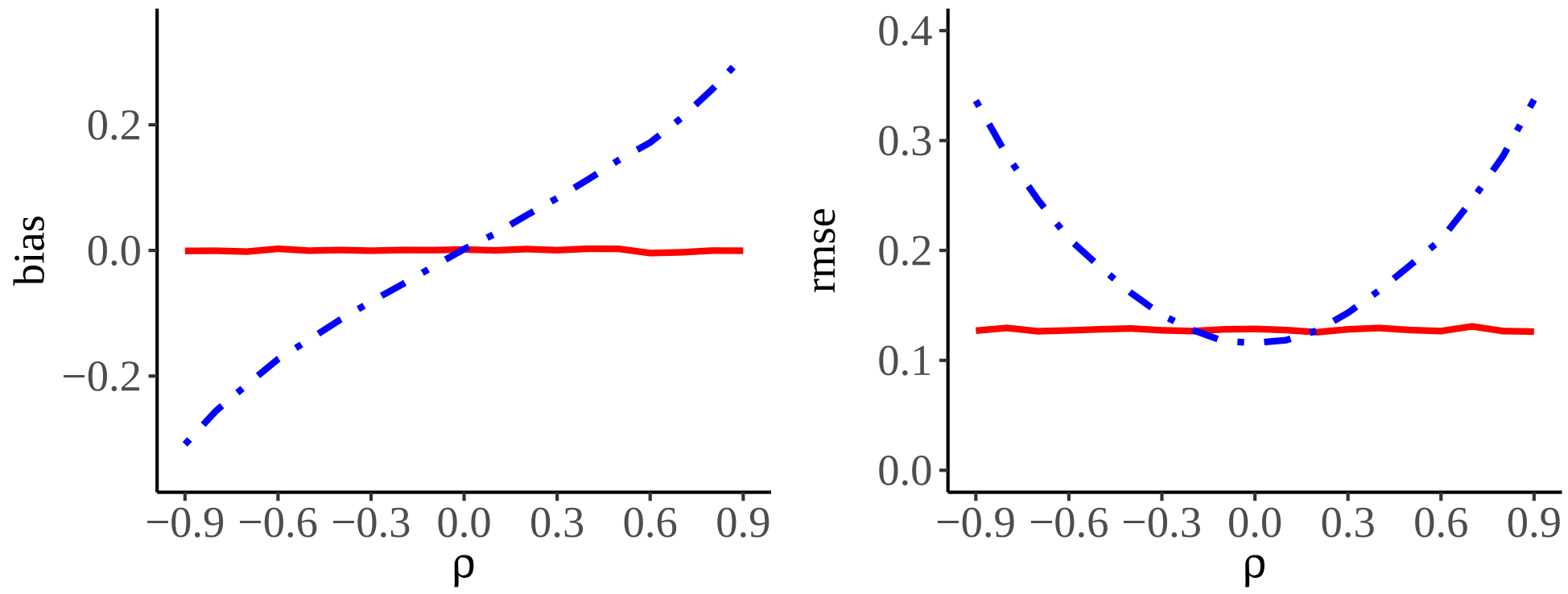

(a) $\alpha_1 = 0$

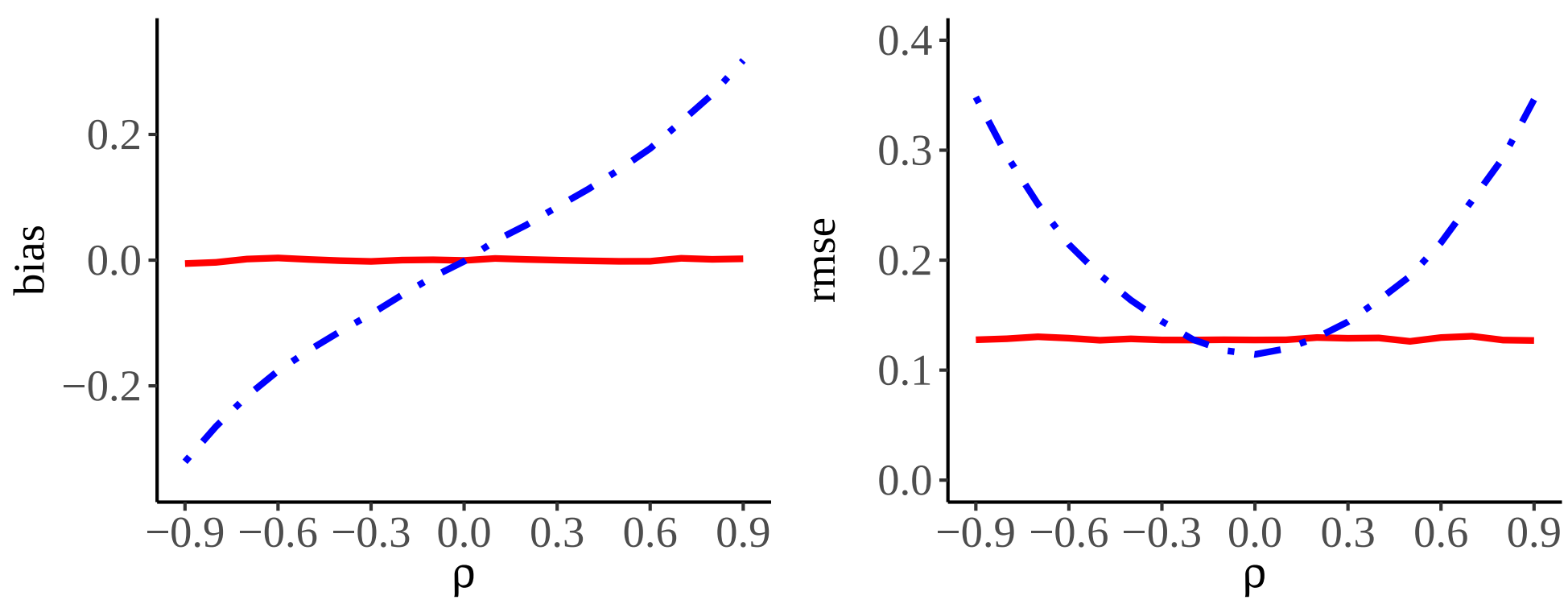

(b) $\alpha_1 = 1.5$

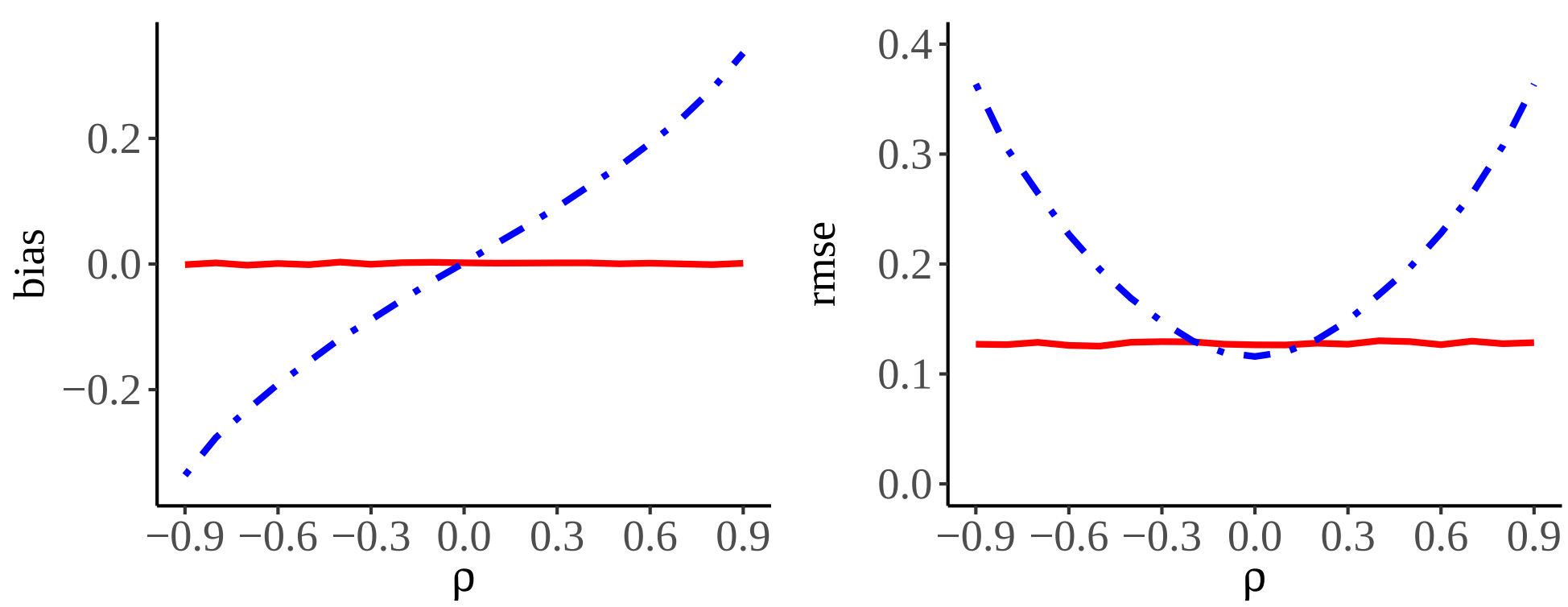

(c) $\alpha_1 = 3$

Figure S6. Empirical bias and RMSE for estimating β across values of ρ, comparing the fixed-effects model (red solid curves) and the random-effects model (blue dashed curves). Panels correspond to different values of the first cluster effect $\alpha_1 = 0$, 1.5, and 3, respectively.

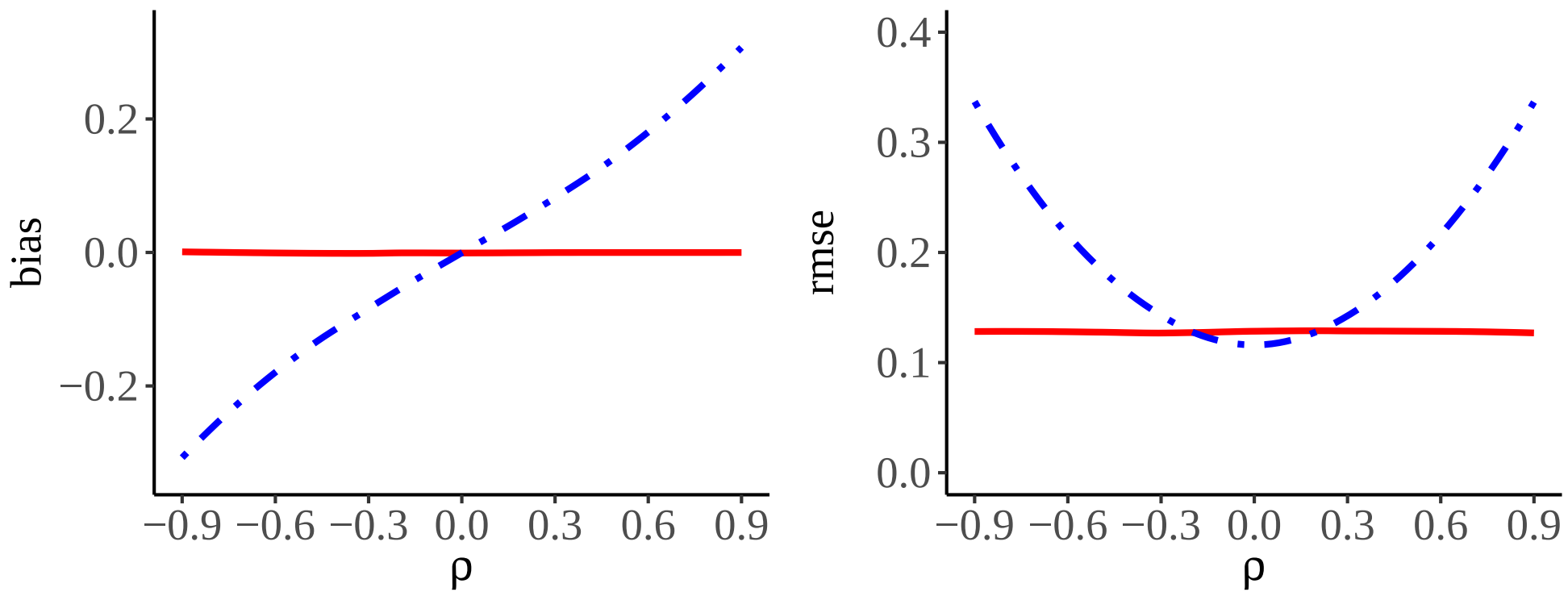

(a) $\sigma_n = 0.1$

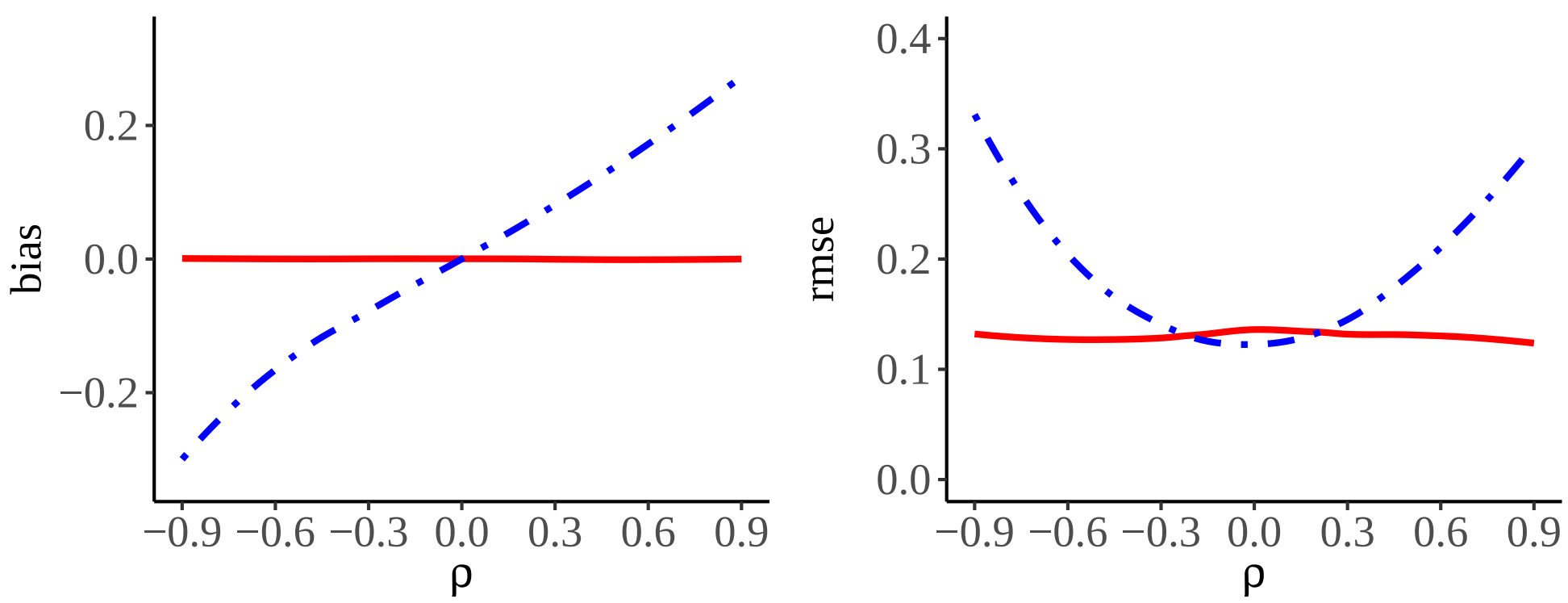

(b) $\sigma_n = 0.6$

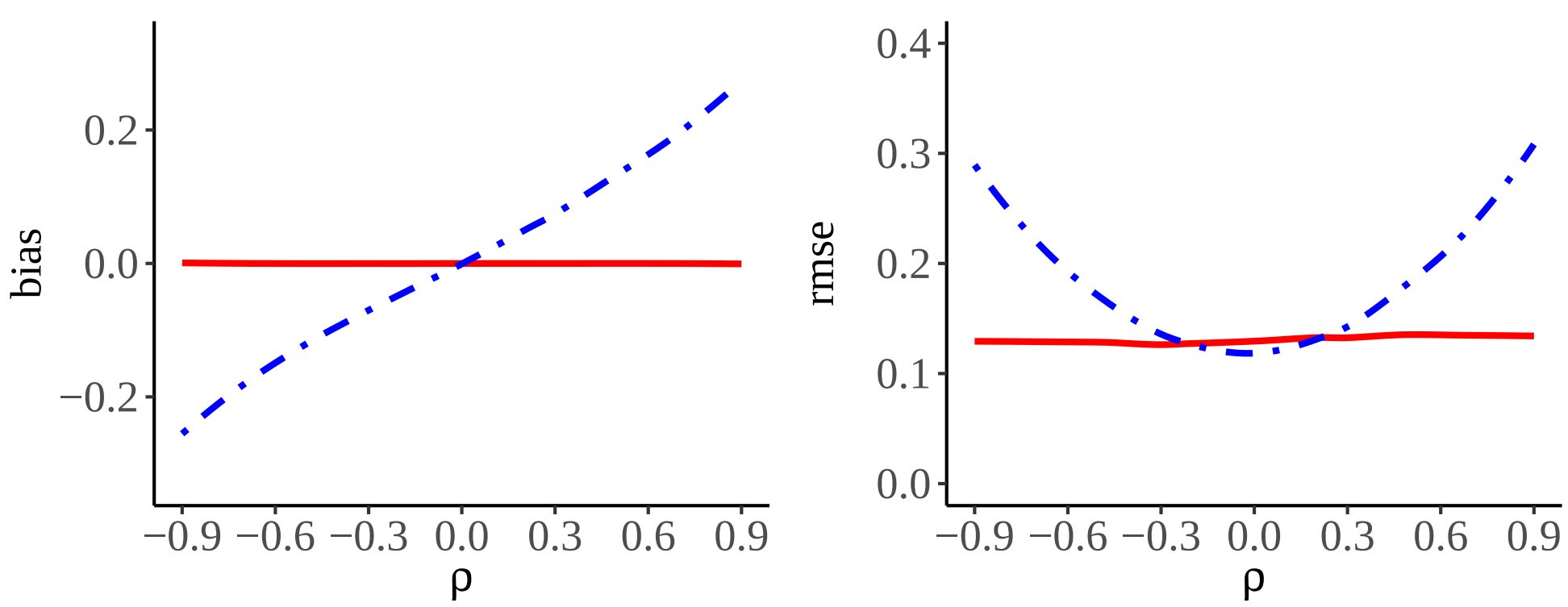

(c) $\sigma_n = 1.0$

Figure S7. Empirical bias and RMSE for estimating β across values of ρ, comparing the fixed-effects model (red solid curves) and the random-effects model (blue dashed curves). Panels correspond to different levels of cluster-size variability with $\sigma_n = 0.1$, 0.6, and 1.0, representing mild, moderate, and high imbalance, respectively.

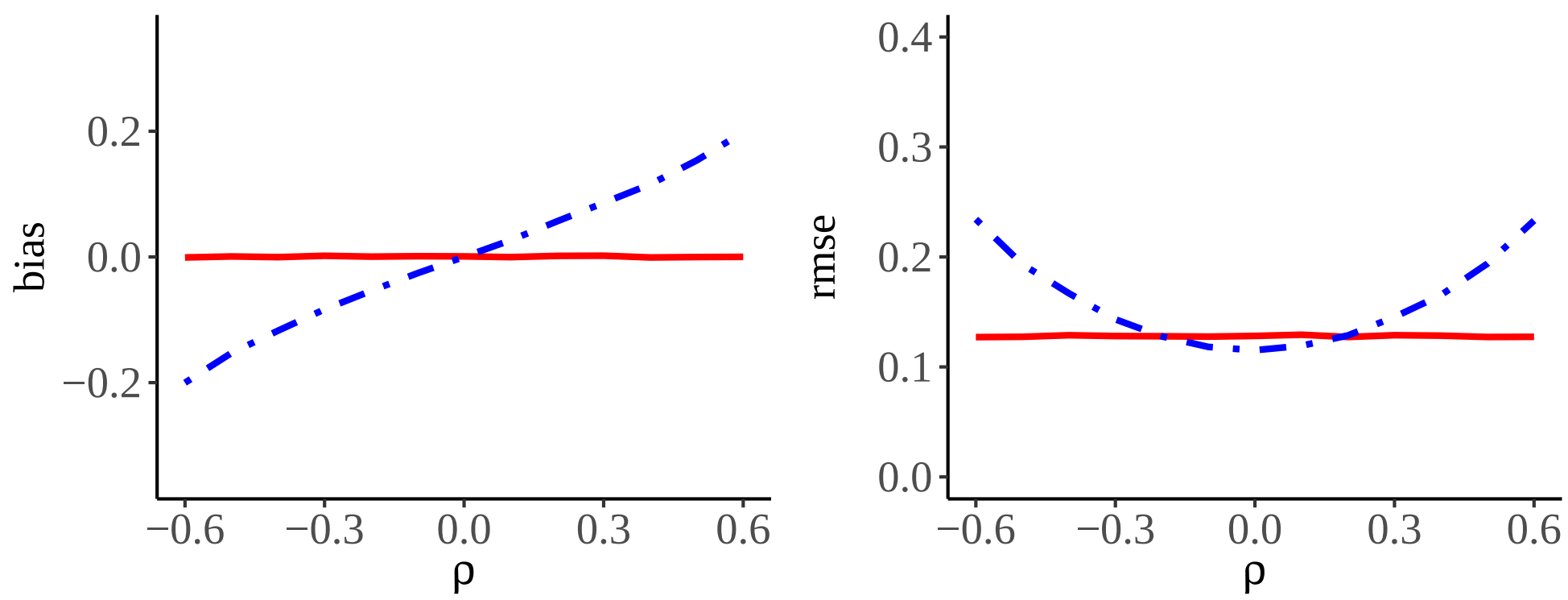

(a) $\rho_\gamma = 0$

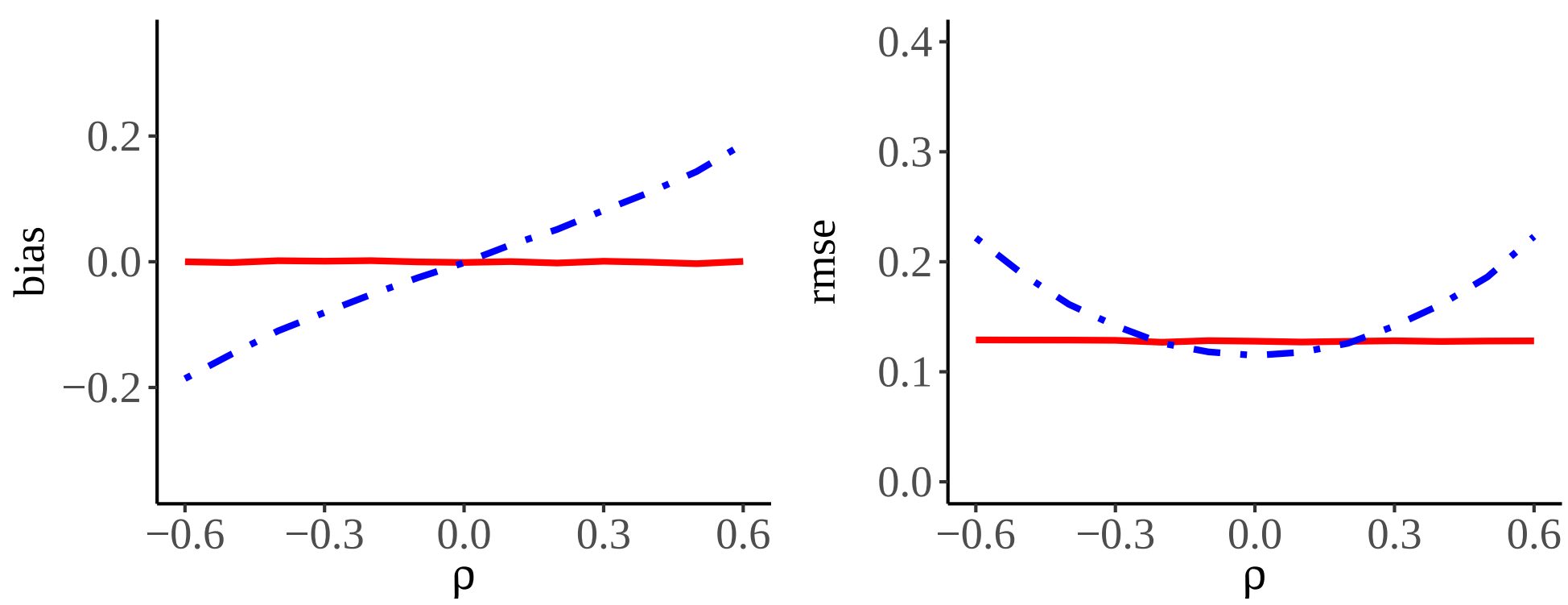

(b) $\rho_\gamma = 0.2$

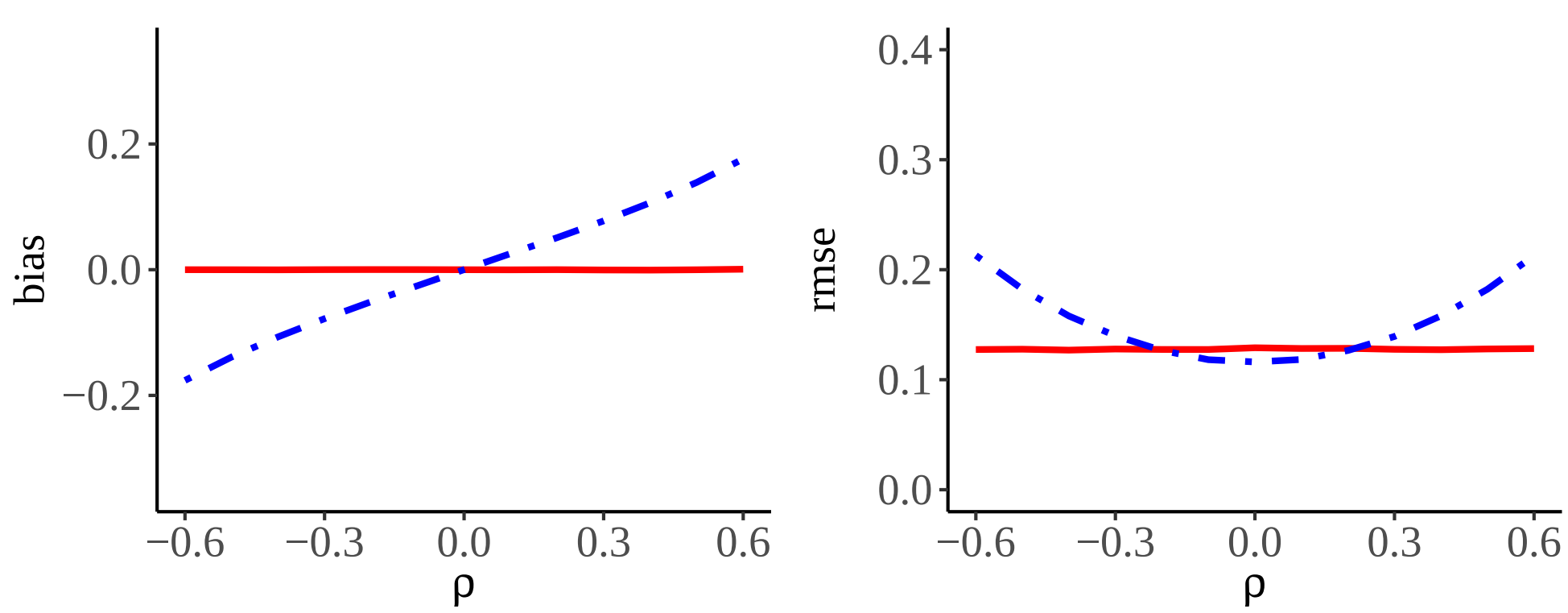

(c) $\rho_\gamma = 0.4$

Figure S8. Empirical bias and RMSE for estimating $\boldsymbol{\beta}$ with $p = 2$ endogenous covariates, averaged across the two regression coefficients. Panels correspond to inter-covariate correlations $\rho_\gamma = 0$, 0.2, and 0.4. Results are shown across values of ρ, comparing the fixed-effects model (red solid curves) and the random-effects model (blue dashed curves).

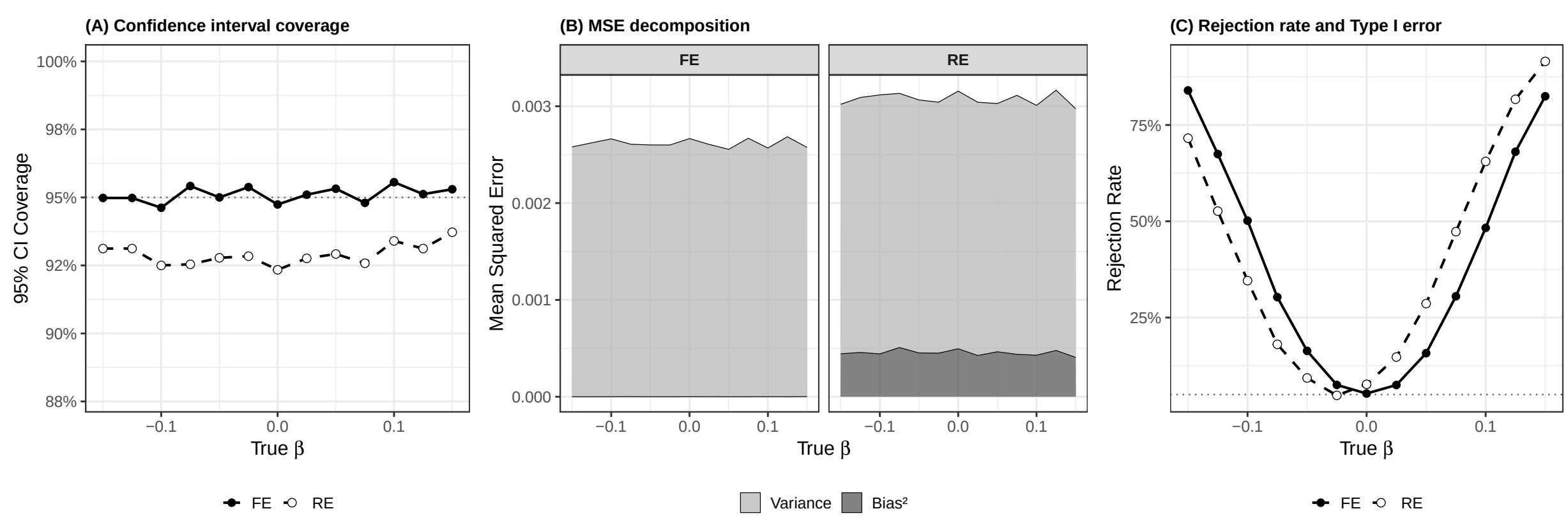

Figure S9. Simulation comparing the inferential quality of the Fixed Effects (FE) and Random Effects (RE) estimators of β as a function of the true effect size ($m = 40$, $n_i = 20$, $\rho = 0.6$, 5,000 replications per grid point). **Panel (A):** Empirical coverage of the 95% Wald confidence interval. The FE estimator (solid line, filled circles) maintains coverage at the nominal 95% level (dotted reference line) across all values of β. The RE estimator (dashed line, open circles) falls to 91–93%, indicating systematic undercoverage driven by the constant positive bias. **Panel (B):** Mean squared error decomposed into variance (light shading) and bias^2 (dark shading). The FE panel contains only variance; the RE panel shows a constant bias^2 floor that elevates MSE above the FE level despite the RE estimator's lower variance. **Panel (C):** Rejection rate for H_0: $\beta = 0$ at the 5% level (dotted reference line). At $\beta = 0$, FE rejects at approximately 5% (correct calibration), whereas RE exhibits inflated Type I error. For positive β, the RE model appears to have higher power, but this apparent advantage is driven by the same bias that inflates Type I error and degrades coverage.
